# Stromal Cell Subsets Show Model-Dependent Changes in Experimental Colitis and Affect Epithelial Tissue Repair and Immune Cell Activation

**DOI:** 10.1093/ibd/izae255

**Published:** 2025-03-18

**Authors:** Zhou Zhou, Jie Su, Bram W van Os, Leonie G Plug, Eveline S M de Jonge-Muller, Lei Brands, Stef G T Janson, Lydia M van de Beek, Andrea E van der Meulen-de Jong, Lukas J A C Hawinkels, Marieke C Barnhoorn

**Affiliations:** Department of Gastroenterology and Hepatology, Leiden University Medical Center, Albinusdreef 2, 2333 ZA, Leiden, The Netherlands; Department of Gastroenterology and Hepatology, Leiden University Medical Center, Albinusdreef 2, 2333 ZA, Leiden, The Netherlands; Department of Gastroenterology and Hepatology, Leiden University Medical Center, Albinusdreef 2, 2333 ZA, Leiden, The Netherlands; Department of Gastroenterology and Hepatology, Leiden University Medical Center, Albinusdreef 2, 2333 ZA, Leiden, The Netherlands; Department of Gastroenterology and Hepatology, Leiden University Medical Center, Albinusdreef 2, 2333 ZA, Leiden, The Netherlands; Department of Gastroenterology and Hepatology, Leiden University Medical Center, Albinusdreef 2, 2333 ZA, Leiden, The Netherlands; Department of Gastroenterology and Hepatology, Leiden University Medical Center, Albinusdreef 2, 2333 ZA, Leiden, The Netherlands; Department of Gastroenterology and Hepatology, Leiden University Medical Center, Albinusdreef 2, 2333 ZA, Leiden, The Netherlands

**Keywords:** stromal cells, inflammatory bowel disease, experimental colitis, fibroblasts, CXCL12, CD90, CD55, podoplanin

## Abstract

**Background:**

Previous work on inflammatory bowel disease (IBD) revealed changes in the abundance of colonic stromal subsets during intestinal inflammation. However, it is currently unknown whether these stromal cell subset changes are also reflected in different IBD mouse models and how commonly used IBD therapies affect stromal cell subset composition.

**Methods:**

Stromal subset markers CD55, C-X-C motif chemokine 12 (CXCL12), podoplanin (PDPN), CD90, and CD73 were analyzed by flow cytometry in 3 mouse models for IBD, namely interleukin (IL)-10 knockout (KO), dextran sulfate sodium-induced, and T-cell transfer model for colitis. Next, the effects of IBD therapies on the stromal subset composition were studied. *In vitro* experiments were performed to study the interaction between stromal cell subsets and epithelial/immune cells.

**Results:**

The colitis-induced changes in the abundance of stromal cell subsets differed considerably between the 3 colitis mouse models. Interestingly, treatment with IBD medication affected specific stromal subsets in a therapy and model-specific manner. *In vitro* experiments showed that specific stromal subsets affected epithelial wound healing and/or T-cell activation.

**Conclusions:**

The relative abundance changes of stromal cell subsets during experimental colitis differ between 3 established colitis models. Treatment with IBD therapies influences stromal subset abundance, indicating their importance in IBD pathogenesis, possibly through affecting epithelial migration, and T-cell activation.

**Key Messages**

**What Is Already Known?**

Single-cell analysis of the inflamed colon of inflammatory bowel disease (IBD) patients revealed different stromal cell subsets and their importance in IBD pathogenesis.

**What Is New Here?**

Our study showed that the stromal subset abundance differed between 3 established colitis mouse models, possibly reflecting the diversity in human IBD. Moreover, our findings demonstrated that clinical treatments for IBD directly impact specific stromal cell subsets.

**How Can This Study Help Patient Care?**

Our study provides important new insights into the role of stromal cell subsets in IBD pathogenesis. Investigating the effects of therapies on stromal cells may provide insights into novel therapeutic targets or the development of new medications.

## Introduction

Inflammatory bowel disease (IBD) is a group of chronic intestinal inflammatory diseases that includes ulcerative colitis (UC) and Crohn’s disease (CD). Patients with IBD suffer from diarrhea, abdominal pain, unintended weight loss, bloody stool, and fatigue.^1^ Although the underlying etiology of IBD is not fully understood, increasing evidence suggests that IBD pathogenesis is a joint result of genetic factors, the host immune system, and environmental factors such as the gut microbiota, disturbing the intestinal homeostasis.^2^ Dysregulated immune homeostasis, caused by damaged epithelial cells and the intestinal microbiome, is one of the main drivers of inflammation.^3^ The most commonly used therapies for IBD, such as thiopurines or antibodies against tumor necrosis factor-α (TNF-α), mainly target immune cells. Unfortunately, 10%-30% of IBD patients do not respond to these treatments and 23%-46% of patients lose initial response over time.^4^

In recent years, a role for stromal cells in IBD pathogenesis has been suggested.^5–7^ Stromal cells are connective tissue cells and are defined as nonhematopoietic, nonepithelial, and nonendothelial cells. The most abundant stromal cells are fibroblasts, of which various subsets exist. Stromal cells can be activated and redistributed to damaged areas where they aid in mucosal repair during inflammatory conditions.^8^ Meanwhile, they affect the immune system through the production of cytokines and chemokines and by direct cell-to-cell contact with leukocytes, especially T cells.^9,10^ Therefore, it is of vital importance to explore the role of stromal cells in the pathogenesis of IBD to find potential stromal targets for therapy.

During the past decade, the heterogeneity of stromal cells has been revealed and various stromal subsets with distinct functions have been identified in colonic and ileum tissues from IBD patients. An inflammation-associated fibroblast subset was present in higher abundance in tissues from UC patients compared to healthy controls.^11^ This subset was enriched for the expression of many genes associated with colitis, including interleukin (*IL*)*-11*, fibroblast activation protein-α (*FAP*), twist family bHLH transcription factor 1 (*TWIST1*), and wingless-type MMTV integration site family member 2 (*WNT2*). Furthermore, the first indications for the role of fibroblast subsets in response to therapy were reported. A subset of IBD patients who did not respond to anti-TNF and corticosteroid therapy showed enrichment for activated fibroblasts in the ulcer bed, which displayed neutrophil-chemoattractant properties.^12^ Finally, it was also reported that oncostatin M (OSM) promotes the inflammatory activity of stromal cells in IBD, and high OSM was associated with resistance to anti-TNF-α therapy in IBD patients.^9^

Besides the identification of inflammation-associated fibroblasts, additional fibroblasts subsets were defined. Kinchen et al. described 4 subsets of stromal cells (S1-S4) by using single-cell RNA sequencing analysis of colon samples from UC patients. The relative abundance of these subsets changed considerably during inflammation, especially for the S2 and S4 subsets. S2 fibroblasts were detected in the crypt niche and essential for colonic epithelial stem cell function, whereas the function of S1, S3, and S4 fibroblasts was not further specified. C-X-C motif chemokine ligand 12 (CXCL12), CD55, and podoplanin (PDPN) were found to identify S1, S3, and S4 subsets, respectively.^13^ CXCL12 is a strong chemotactic for lymphocytes^14,15^ and CD55 is a regulator of the complement system on the cell surface.^16^ Podoplanin is a transmembrane glycoprotein involved in fibroblast migration in multiple cancers.^17–20^ Other interesting markers to define subpopulations of fibroblasts are CD90 and CD73. CD90^+^ colonic stromal cells are reported as immunosuppressors and are among the key regulators of acute and chronic inflammation.^21^ CD73 is a glycosylphosphatidylinositol-linked cell surface enzyme, suppressing immune activation via generating adenosine.^22^ Taken together, several human fibroblast subsets have been described with a potential role in IBD pathogenesis and response to therapy.

In this work, 3 broadly used experimental colitis mouse models (dextran sulfate sodium (DSS)-induced colitis, IL-10 knockout (KO) mice, and T-cell transfer mouse model) were used to unravel changes in the stromal component during the course of inflammation. Based on the subsets identified in human IBD, CXCL12, CD55, PDPN, CD90, and CD73 were selected as markers to investigate changes in stromal subset abundance during intestinal inflammation. In addition, the effects of 3 clinically applied IBD therapies (thiopurines/anti-TNF/anti-p40) on stromal subset abundance were assessed. Finally, the interaction between stromal cell subsets and epithelial/immune cells was studied. Our data illustrate that the stromal subset composition is differentially altered in the 3 mouse IBD models and show model-dependent alterations in response to commonly used IBD therapies.

## Materials and Methods

### Animal Experiments

All the experiments were approved by the Dutch Animal Ethics Committee and the Central Authority for Scientific Procedures on Animals (Permit number: 116002017860). Animals were housed in ventilated cages and were given drinking water and food ad libitum.

Three mouse models for IBD were established: the IL-10 KO, DSS-induced colitis, and T-cell transfer model. For the IL-10 KO model, B6.129P2-Il10tm1Cgn/J mice (IL-10^−/−^) were bred in the animal breeding facility of the Leiden University Medical Center (LUMC). Eight to 16-week-old female mice (*n* = 6-7 mice/group) received 200 ppm piroxicam (Sigma, Zwijndrecht, The Netherlands) homogenized in their food for 11 days to accelerate colitis development as described before.^23^ For the DSS model, 1.5% DSS (MV36,000-50 000 kDa, MP Biomedicals, IllKirch, France) was supplied to the drinking water of 8-week-old female C57BL/6 Jico mice (*n* = 10 mice/group, Charles River Laboratories, The Netherlands) for 7-10 days. Mice were sacrificed on days 7-10. Monitoring for both models included daily body weight, stool consistency, and colonic hemorrhage. On sacrifice day, mice underwent an endoscopy under isoflurane anesthesia to determine the murine endoscopic index of colitis severity (MEICS).^24^ Following endoscopy, mice were sacrificed and organs were collected for further analysis. The T-cell transfer model was established as previously described.^25^ In short, 8-week-old female C57BL/6 Jico donor mice (Charles River Laboratories) were euthanized, and their spleens were harvested. Next, 500 000 CD4^+^CD45RB^high^ T cells were sorted from splenocytes using flow cytometry and intraperitoneally injected into 9-week-old B6.129S7-Rag1tm1Mom/J (Rag^−/−^) mice (*n* = 7-10 mice/group, own breeding). Mice were monitored every 3 days. From week 3, the MEICS evaluated every 2 weeks. Mice were sacrificed on day 49, after which colon tissues were collected for flow cytometry and histology analysis.

To investigate the effects of IBD therapies on the stromal subset composition, anti-mouse TNF (IgG2a mAb, clone CNTO5048), anti-mouse IL-12/23-p40 (IgG2a mAb, clone CNTO3913, both kind gifts from Janssen Research & Development), and 6-thioguanine (6-TG, Sigma), were administered to T-cell transfer colitis, IL-10 KO, and DSS-induced colitis mice, respectively. The choice of medication was based on previously reported clinical effects of the drug/model combinations.^26–28^ T-cell transfer colitis mice were treated with 50 µg anti-mouse TNF, or isotype control mAbs (IgG2a mAb, clone CNTO6601) 2 times per week via intraperitoneal injections for about 3 weeks (*n* = 3-5 mice per group). Treatment was started when colitis was evident as defined by MEICS score >2 and mice were sacrificed on day 47. Interleukin-10 KO mice received 200 µg anti-mouse IL-12/23-p40 or isotype control mAbs via intraperitoneal injection from day 11 onwards (*n* = 4 mice/group). Mice were treated twice weekly for about 3 weeks. The MEICS score was evaluated on days 9, 11, 14, 24, and 29, and mice were sacrificed on day 29. For the DSS model, C57BL/6 mice were treated via oral gavage with 2 mg/kg 6-TG in tap water on days 3, 5, 6, 7, and 8 (*n* = 10 mice/group). Endoscopy was performed on day 10 to assess the MEICS score. Length and weight of the colon were determined and colon tissue was collected for flow cytometry and histological analysis.

### Flow Cytometry

Colonic tissues were minced with scissors and incubated in 375 µg/mL Liberase TL solution (Sigma) dissolved in Dulbecco’s Modified Eagle Medium (DMEM)/F12/Glutamax (Thermo Fisher Scientific, Paisley, UK) containing 10% fetal calf serum (FCS; Thermo Fisher Scientific), for 30 minutes at 37 °C. To obtain single cells, the suspension was filtered through Falcon Cell Strainers with 70-µm pore size (Corning, Durham, USA) and washed in flow cytometry buffer (FCB; 0.5% bovine serum albumin (BSA; Sigma), 0.02% NaN_3_ in PBS (Pharmacy LUMC, Leiden, The Netherlands).^29^ Cells were stained with surface antibodies described in Supplementary Table S1 for 45 minutes at room temperature (RT) and then fixed with fixation concentrate (Thermo Fisher Scientific) for 1 hour at RT. Permeabilization buffer (Thermo Fisher Scientific) was used to permeabilize cells to stain for the intracellular marker CXCL12-APC (R&D, MN, USA), followed by detection of CD31 (which was a biotin-labeled antibody) with the secondary antibody Brilliant Violet 605 Streptavidin for 45 minutes at RT. Cells were washed with FCB and measured on the Fortessa flow cytometer (BD Bioscience, Vianen, The Netherlands). Flow cytometry data analysis was performed using Flowjo (version 10.0.6; FlowJo, data analysis software) and/or FCSexpress 7.0. Dead cells, hematopoietic cells (CD45^+^), endothelial cells (CD31^+^), and epithelial cells (Epcam^+^) were excluded to obtain the stromal cell population. The applied gating strategy is specified in Supplementary Figure S1. Uniform manifold approximation and projection (UMAP) was generated using FCSexpress 7.0

### Analysis of Single-Cell RNA Sequencing (scRNA-seq) Datasets

To explore the stromal makers’ expression pattern in human stromal cells, the publicly available data GSE114374,^13^ consisting of colon samples from 2 healthy and 2 UC patients, and GSE134809,^30^ including 11 paired resection ileal samples from the inflamed and uninflamed areas of CD for the scRNA-seq were retrieved from the gene expression omnibus (GEO) database. The cell gene expression matrices for further analysis by using the R package Seurat (version 4.0.3) were used.^31^ After filtering low-quality cells (default setting, min.cells = 3, min.features = 200), reciprocal principal component analysis (PCA) was used for data integration, nearest-neighbor graphs using the top 30 dimensions of the PCA reduction were calculated and the clustering analysis was applied with a resolution of 0.2 using UMAP. The “FindMarkers” function (default setting, min.pct = 0.25, log_2_Fold change threshold = 0.25) was applied to identify the markers, including *CXCL12*, *CD55, CD90, PDPN*, and *CD73* that defined each cluster, which automatically, via differential expression analysis, compared a cluster against all others. Cluster annotations were based on canonical marker genes. In GSE114374, whole biopsies were dissociated into single cells and depleted for EPCAM^+^, CD45^+^, and CD235a^+^ cells. Thereafter exclusion markers, including *PECAM1* (endothelial cells), *S100B* (glial cells), *RGS5* (pericytes), and *SDC1* (plasma cells), were used to define stromal cells.^13^ In GSE134809, markers for fibroblasts (*CCL13* and *CCL8*) and activated fibroblasts (*CXCL8*, *CXCL3*, *CXCL1*, and *CXCL6*) were used to identify stromal cells.^30^ To specify the stromal markers’ expression pattern at different stages of inflammation in mice, the publicly available dataset GSE148794,^32^ in which mice were treated with 1.5% DSS for 6 days and colon samples were collected at different time points (days 0, 3, 6, 9, 12, and 15) for the following scRNA-seq, was retrieved from GEO database for analysis. In GSE148794, the stromal cluster was identified based on the expression of *Col1a1*, *Pdgfra*, and *Spon2*.^32^

### Constructs, Lentiviral Transduction, Generation, and Validation of Transgenic Cell Lines

Third-generation packaging vectors and HEK293T cells were used for the generation of lentiviral particles as described before.^33^ Knockdown (KD) constructs for CXCL12, CD55, and CD90 were acquired from the Mission TRC1 shRNA library (Sigma) with target sequences as indicated in Supplementary Table S2. The lentiviral cDNA expression vectors were generated through Gateway cloning, using the gene entry vector (pDNR223 backbone) from the human open reading frames library (Sigma) and pLex307 (addgene: #41392) as the destination vector.^34^ Knockdown murine fibroblasts 3T3-J2 cells were selected with 2 µg/mL of puromycin (Sigma). Knockdown efficiency was confirmed by quantitative polymerase chain reaction (qPCR) and flow cytometry analysis.

### RNA Isolation and qPCR

RNA isolation and qPCR analysis were performed as described before.^35^ In short, RNA was extracted using the NucleoSpin RNA isolation kit (Macherey-Nagel, Düren, Germany) according to the manufacturer’s instructions. The RNA concentration was measured with NanoDrop 1000 Spectrophotometer (Thermo Fisher Scientific). Complementary DNA (cDNA) was synthesized by using RevertAid First strand cDNA synthesis kit (Thermo Fisher Scientific). Quantitative polymerase chain reaction was performed with SYBR Green Master mix (Bio-Rad Laboratories, Nazareth, Belgium) using the iCycler Thermal Cycler and iQ5 Multicolour Real-Time PCR Detection System (Bio-Rad). Target genes were amplified using specific primers (Supplementary Table S3). Target gene expression levels were normalized to the housekeeping gene *β-actin* or *Ptp4a2*. The ΔCt method was applied to calculate the levels of gene expression, relative to the reference gene.^36^

### Cell Culture, *In Vitro* Stimulation, and KD-Conditioned Medium Generation

Murine fibroblasts 3T3-J2 and mouse colonic epithelial cells CT26 were cultured in (DMEM)/F12/Glutamax with 10% FCS, 100 IU/mL penicillin/streptomycin (P/S; Thermo Fisher Scientific) at 37 °C, 5% CO_2_. 3T3 cells were stimulated with 50 ng/mL mouse CXCL12 recombinant protein (Thermo Fisher Scientific) for 6 hours and were then collected for qPCR analysis. 3T3 cells with indicated shRNA knockdown constructs were grown to subconfluence and incubated with serum-free DMEM for 3 days. Conditioned medium (CM) was harvested and centrifuged to remove cellular debris, aliquoted, and stored at −20 °C until use. Primary murine intestinal fibroblasts were isolated from murine colons. To isolate fibroblasts, the tissue was washed with HBSS^−/−^ (Gibco) and treated with a 3:1 collagenase type II (Thermo Fisher Scientific) and dispase II (Roche, Mannheim, Germany) mix. The mixture was incubated at 37 °C for 1.5 hours and vortexed every 30 minutes. The single-cell suspension was collected and cultured in DMEM/F12/Glutamax with 10% FCS, 100 IU/mL P/S, 2.5 µg/mL fungizone, and 50 µg/mL gentamicin (all Thermo Fisher Scientific) at 37 °C, 5% CO_2_ until outgrowth of fibroblast-like cells was observed. Fibroblasts were used between passages 5 and 10 and seeded in 6-well plates (Corning) at 100 000 cells per well. After 24 hours, fibroblasts were treated with 100 µM 6-TG or 10 µg/mL anti-TNF or 20 µg/mL anti-p40 for 48 hours. After treatment, fibroblasts were collected for flow cytometry analysis as described above.

### Colony Formation and Wound Healing Assays

3T3-J2 or CT26 cells (murine epithelial cells) were seeded in 12-well plates (Corning) at 1000 cells per well. For proliferation assays, after 24 hours cells were stimulated with fibroblast CM supplemented with 10% FCS and 1% P/S. The medium was refreshed once weekly. After 2-3 weeks, cells were fixed with 2% paraformaldehyde (PFA) and stained with 0.05% crystal violet (Sigma) to identify the colonies. Pictures were analyzed with ImageJ (US National Institute of Health, USA), and the percentage-stained area was determined.

For the wound healing assay, 3T3-J2 or CT26 cells were seeded in 48-well plates (Corning) at a density of 25 000 cells per well. When reaching confluence, a scratch was made with a p200 pipet tip. Debris was removed by washing the cells once and cells were stimulated with control medium (FCS-free) or fibroblast CM. Cells were incubated at 37 °C in the Biotek Cytation5 equipped with Biotek CO_2_ gas controller (Biotek Instruments, Inc, Winooski, VT, USA). Pictures were automatically obtained at indicated time points. The scratch area of each image was measured with ImageJ.


$$\begin{array}{l} Wound\;closure\;\%=\frac{\left(A_{0}\;-\;A_{t}\right)}{A_{0}}\;\times \;100\%, \end{array}$$

where *A*_0_ and *A*_*t*_ are the scratch area measured immediately and *t* hours after scratching, respectively.

### CoCulture of Fibroblasts and CD4^+^ T Cells

OT-II transgenic mice (B6.Cg-Tg(TcraTcrb)425Cbn/J) were obtained from the LUMC Department of Immunology. Spleens were harvested and cells were passed through a 70-µm cell strainer. Erythrocytes were lysed with lysis buffer (NH4Cl: 8.4 g/L; KHCO_3_: 1 g/L, pH = 7.4 ± 0.2, Pharmacy LUMC). Splenocytes were labeled with 5 µM carboxyfluorescein succinimidyl ester (CFSE; Thermo Fisher Scientific) activated with 2 mg/mL ovalbumin (OVA) peptide 323-339 (GenScript, Rijswijk, The Netherlands) for 4 hours, and cocultured with 3T3 fibroblasts at a 1:10 ratio in Iscove’s Modified Dulbecco’s Medium (IMDM; Thermo Fisher Scientific) in the presence of 10% FCS, 1% P/S, and 25 μM 2-mercaptoethanol (Sigma). After 3 days of coculture, cells were collected and stained with the antibodies listed in Supplementary Table S4 for 45 minutes on ice. Carboxyfluorescein succinimidyl ester signal was measured on the Fortessa flow cytometer and data analysis was performed as described above.

### Tissue Processing and Histological Analysis

Tissues were formalin-fixed, processed in a tissue processor (Leica, Amsterdam, The Netherlands), and subsequently embedded in paraffin. For hematoxylin and eosin staining, slides were deparaffinized and rehydrated and then stained with hematoxylin (Sigma-Aldrich) for 5 minutes. After washing with tap water, the slides were stained with eosin (Sigma-Aldrich) for 1 minute and dehydrated before being mounted with Entellan (Merck KGaA, Darmstadt, Germany). Images were made with an Olympus BX52 microscope.

Immunohistochemistry (IHC) and immunofluorescent stainings (IF) were performed as described before.^7,15^ In short, paraffin sections (4 µm) were deparaffinized and rehydrated, after antigen retrieval was performed by 10 minutes boiling in 0.01 M sodium citrate buffer, pH 6.0. Slides were then incubated with primary antibodies, rabbit anti-CD3 (1:1000; DAKO Netherlands BV, Herelee, Belgium), rabbit anti-CD4 (1:400; Cell Signaling, Massachusetts, USA), rabbit anti-CD8 (1:400; Cell Signaling), rat anti-FOXP3 (1:400; eBioscience Ltd, Hatfield, UK), mouse anti-CXCL12 (1:400; R&D), mouse anti-CD90 (1:1000; Proteintech, Rosemont, USA), and rat anti-CD45 (1:400; eBioscience) diluted in 1% bovine serum albumin (BSA) in PBS overnight at 4 °C. Afterward, the appropriate secondary biotinylated antibodies (Agilent Technologies, CA, USA) or anti-rat-Alexa 488 (Thermo Fisher Scientific) and anti-mouse-Alexa 594 (Thermo Fisher Scientific) were applied (1:200, 30 minutes at RT). This was followed by incubation with Vectastain (Vector Laboratories, CA, USA) for 30 minutes and visualization with Dako Liquid DAB + Substrate Chromogen System (Agilent Technologies) for 10 minutes at RT for IHC stainings. The slides were counterstained with hematoxylin, dehydrated, and mounted. Images were made with an Olympus BX52 microscope. For the quantification, 5 random pictures per slide were obtained. The staining was analyzed by quantifying the number of DAB-positive cells with Image J software (https://imagej.net/). For IF stainings, Prolong Gold Antifade reagent with 4’,6-diamidino-2-phenylindole (DAPI) (Invitrogen, Waltham, USA) was applied. Images were made with Widefield Leica DM6B2 (Leica Microsystems, Germany).

### Statistical Analysis

Data were presented as means ± SD from representative experiments or independent biological replicates as indicated in the figure legend. Nonpaired or paired Student *t*-test, or Mann–Whitney *U* tests were used to compare 2 groups where appropriate. All analyses were performed using GraphPad Prism Software (San Diego, CA, USA). *P* values of .05 or less were considered statistically significant.

## Results

### Stromal Subset Abundance Is Different in Three Experimental Colitis Models

**Figure 1.**
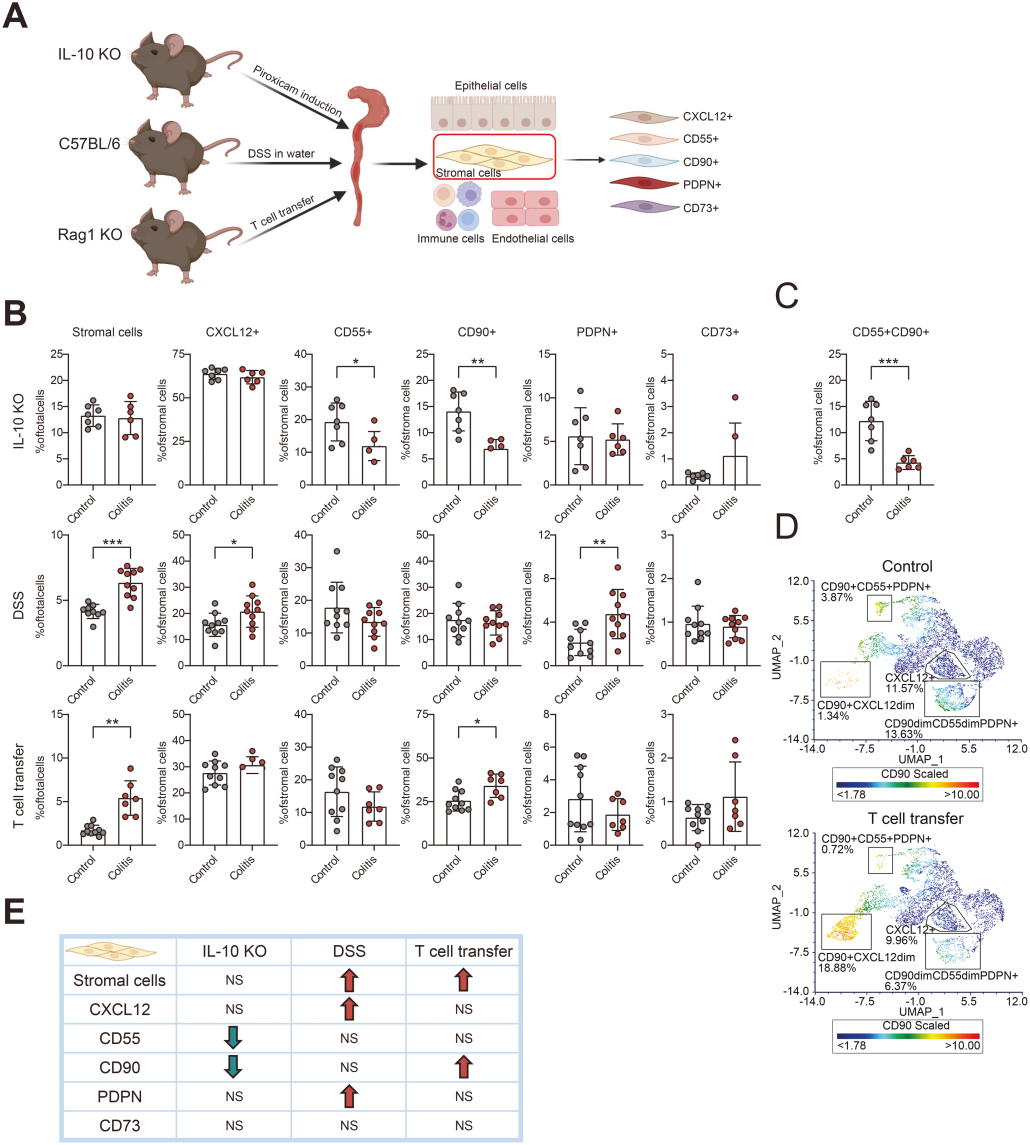
Different stromal subset abundance in 3 experimental colitis models. A, Schematic overview of the experiment set-up, 3 different experimental colitis models were used. Epithelial cells, immune cells, and endothelial cells were excluded. Stromal cell subsets were identified by stromal markers. B, Bar graph of the percentage of stromal cells in 3 colitis mouse models. In IL-10 knockout (KO) colitis, control, *n* = 7, colitis, *n* = 6; in dextran sulfate sodium-induced colitis, control, *n* = 10, colitis, *n* = 10; in T-cell transfer colitis, control, *n* = 10, and colitis, *n* = 7. C, Bar graph of the percentage of stromal cells in IL-10 KO colitis. D, Uniform manifold approximation and projection (UMAP) plot of stromal cells in T-cell transfer colitis. E, Overview of the trend of percentage of stromal subsets in 3 colitis mouse models. The direction of the arrows indicate a significant increase or decrease (*P* ≤ 0.05). The data are presented as the mean ± SD. Nonpaired two-tailed *t*-test or Mann–Whitney *U* test was performed. *P ≤ 0.05, ***P ≤ 0.001, ****P ≤ 0.0001. NS, no significance.

To compare stromal subset abundance in 3 different models for experimental colitis, colitis was induced and inflammation was evaluated (Figure 1A). Colitis was successfully induced in all 3 models (IL-10 KO, DSS-, and T-cell transfer–induced colitis) as determined by loss of body weight (Supplementary Figure S2A), increased MEICS score (Supplementary Figure S2B), and histologic features of colitis (Supplementary Figure S2C). Mice were sacrificed during the active inflammation phase at days 7-10 in the DSS model, day 11 in the IL-10 KO mice, and day 49 in T-cell transfer mice. Flow cytometry analysis confirmed a strong increase in the percentage of CD45^+^ cells in all 3 colitis groups (Supplementary Figure S2D). Evaluation of total stromal cell content in the colon by flow cytometry revealed higher stromal abundance upon colitis in both the T-cell transfer and DSS models while the total number of stromal cells remained unchanged in the IL-10 KO mice (Figure 1B). Within the stromal cell population, 5 distinct stromal subset markers, CXCL12, CD90, CD55, PDPN, and CD73, were analyzed. In all models, the small population with CD73^+^ stromal cells did not change upon colitis induction. In the IL-10 KO mice, the percentage of CD55^+^ and CD90^+^ stromal cells was significantly decreased (Figure 1B). Interestingly, a substantial part of these cells were positive for both CD55 and CD90 (Figure 1C). During DSS-induced colitis, the percentage of CXCL12^+^ and PDPN^+^ stromal cells was increased compared to healthy control mice (Figure 1B). Changes in PDPN^+^ stromal cells showed high variation between individual experiments since the abundance of PDPN^+^ stromal cells was decreased in our other DSS-induced colitis experiment. Interestingly, analysis of the scRNA-seq dataset GSE148794, in which scRNA-seq was performed at days 0, 3, 6, 9, 12, and 15 during DSS-induced colitis and recovery, revealed, in accordance with our results, that the abundance of *Cxcl12*^*+*^ and *Pdpn*^*+*^ stromal cells increased during the inflammation phase. However, the percentage of *Cd90*^*+*^ and *Cd55*^*+*^ stromal cells reached their peak percentages already 3 days after induction of DSS and began to decrease thereafter (Supplementary Figure S3A-F). These data show that stromal subsets exhibit dynamic alterations during the progression and recovery phase of the disease. In T-cell transfer–induced colitis, the percentage of CD90^+^ stromal cells was significantly increased. UMAP analysis showed a substantial increase in a specific CD90^+^ subpopulation, representing 1.34% of the stromal content in control mice and 18.88% after colitis induction (Figure 1D). Interestingly, a population of CD90^+^CD55^+^PDPN^+^ cells was significantly decreased (3.87% vs 0.72%) next to another CD90^dim^ population co-expressing CD55^+^PDPN^+^ (13.63% vs 6.37%, Figure 1D). Altogether, these data (summarized in Figure 1E) show that the composition of stromal cell subsets differs between 3 established colitis mouse models, possibly reflecting different subtypes and phases of human IBD.

**Figure 2.**
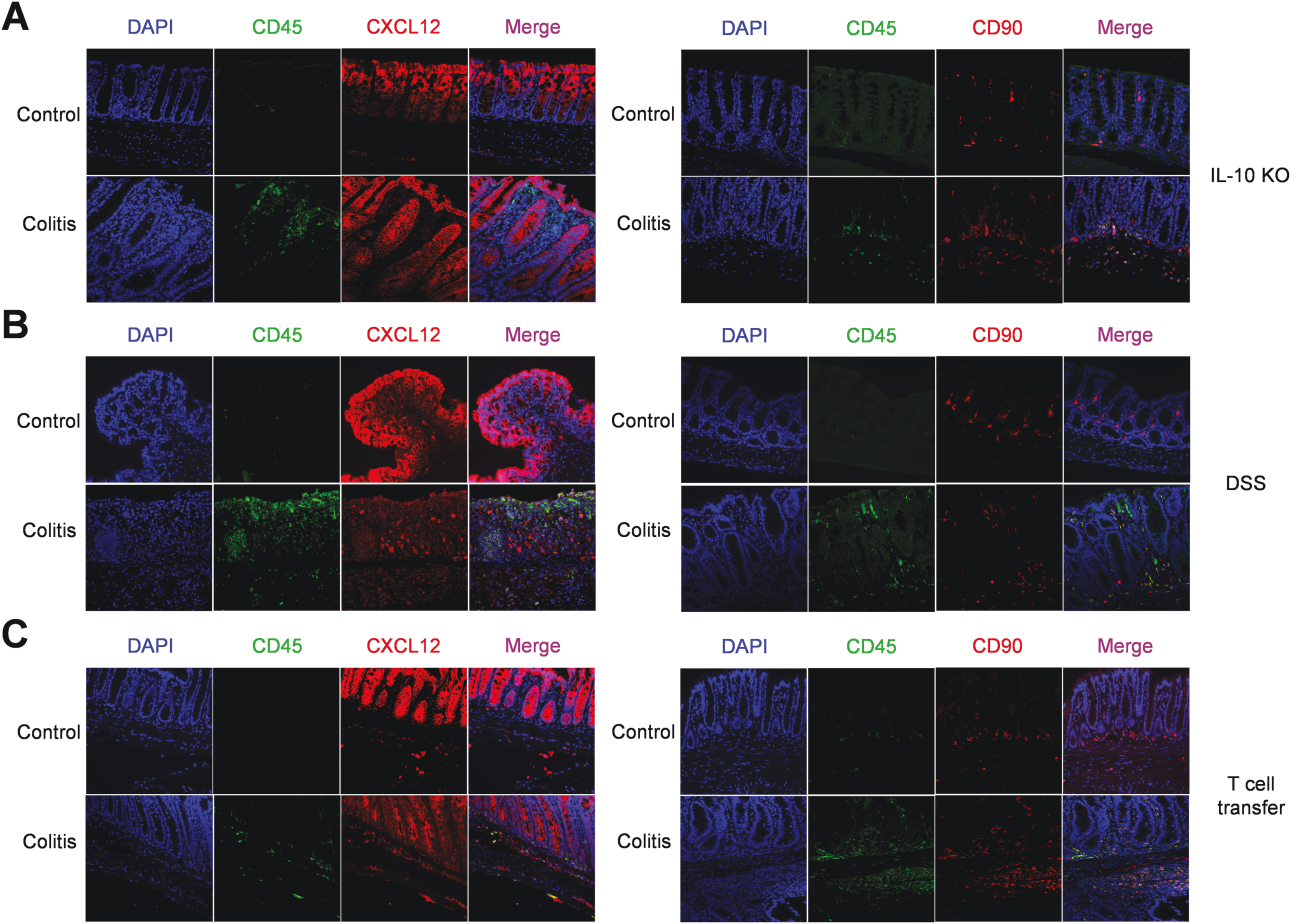
Redistribution of stromal subsets in experimental colitis models. Images of immunofluorescent staining of CXCL12 (left) and CD90 (right) together with CD45 in mice colon of IL-10 knockout colitis (A), dextran sulfate sodium-induced colitis (B) and T cell transfer colitis (C). Magnification 40×.

To investigate the spatial distribution of identified stromal subsets within the colon, we performed immunofluorescent stainings. Our data revealed distinct patterns of staining for stromal markers in different colitis models. In all 3 models, we observed the expression of CXCL12 on epithelial cells (Figure 2). In IL-10 KO colitis mice, few CXCL12^+^ fibroblast-like cells were identified pericryptical (Figure 2A). In IL-10 KO mice without colitis, no CXCL12^+^ fibroblast-like cells were found pericryptical, while some could be identified in the submucosal area (Figure 2A). In the DSS colitis, CXCL12^+^ fibroblasts were present between immune cells in the inflamed mucosa (Figure 2B). In healthy BL/6 mice, CXCL12^+^ fibroblast-like cells were observed both between crypts and in the submucosal area (Figure 2B). In T-cell transfer model, CXCL12^+^ fibroblasts were mainly distributed in the submucosal area in both colitis and control mice (Figure 2C). Interestingly, CD90^+^ fibroblasts were found to be distributed pericryptical and predominantly localized at the basal part of the crypts, as well as in the muscularis mucosa in both IL-10 KO mice with and without colitis (Figure 2A). In DSS colitis, CD90^+^ fibroblasts were primarily located beneath the crypts, while in healthy control mice, these cells appeared to reside between the crypts (Figure 2B). CD90^+^ fibroblasts were also mainly located beneath the crypts in T-cell transfer control mice, while in colitis mice, these cells were distributed in the submucosal area (Figure 2C). These observations suggest that some stromal subsets undergo a dynamic redistribution during the course of disease development.

### Comparison of Human and Murine Stromal Subset Abundance in IBD

**Figure 3.**
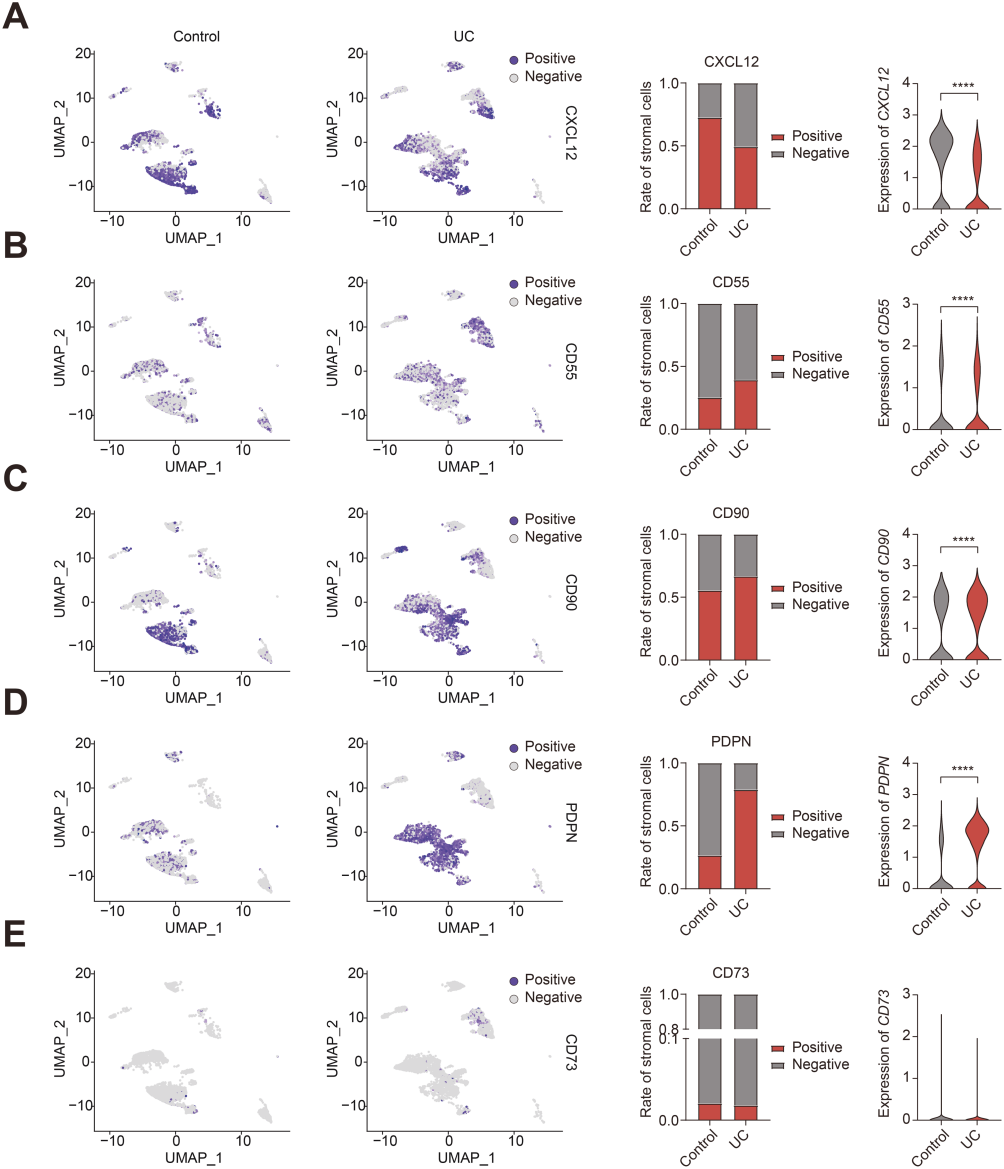
Composition of stromal subsets abundance in human ulcerative colitis (UC) patients. A, Uniform manifold approximation and projection (UMAP) plot of *CXCL12*^+^ stromal cells (left), rate of *CXCL12*^+^ positive and negative stromal cells (middle, Fisher’s exact test was performed to assess statistical significance), and violin plots of expression of *CXCL12* in stromal cells (right, Mann–Whitney *U* test was performed) in datasets GSE114374. B, UMAP plot of *CD55*^+^ stromal cells (left), rate of *CD55*^+^ positive and negative stromal cells (middle), and violin plots of expression of *CD55* in stromal cells (right) in datasets GSE114374. C, UMAP plot of *CD90*^+^ stromal cells (left), rate of *CD90*^+^ positive and negative stromal cells (middle), and violin plots of expression of *CD90* in stromal cells (right) in datasets GSE114374. D, UMAP plot of *PDPN*^+^ stromal cells (left), rate of *PDPN*^+^ positive and negative stromal cells (middle), and violin plots of expression of *PDPN* in stromal cells (right) in datasets GSE114374. (E) UMAP plot of *CD73*^+^ stromal cells (left), rate of *CD73*^+^ positive and negative stromal cells (middle), and violin plots of expression of *CD73* in stromal cells (right) in datasets GSE114374. Positive and negative stromal cells are indicated in healthy controls (UMAP plot, left), and UC (UMAP plot, right) colon. Exclusion markers, including *PECAM1*, *S100B*, *RGS5*, and *SDC1*, were used to exclude the other cell types. ****P ≤ 0.0001.

In order to compare the stromal subsets of different mouse models to human IBD patients, we evaluated the abundance and expression of these subset markers in published human IBD intestine-derived stromal cell datasets. By analyzing the scRNA-seq dataset GSE114374 (colon biopsies from 2 healthy and 2 UC patients),^13^ we found that the percentage of stromal *CXCL12*^*+*^ cells was decreased (Figure 3A), whereas stromal *CD55*^*+*^ (Figure 3B), *CD90*^*+*^ (Figure 3C), and *PDPN*^*+*^ (Figure 3D) cells were increased in UC patients compared to healthy controls. The abundance of stromal *CD73*^*+*^ showed no significant changes (Figure 3E; Supplementary Table S5). With regard to the expression of these markers in stromal cells, the expression of *CXCL12* and *CD55* was decreased, whereas *CD90* and *PDPN* were increased (Figure 3). In GSE134809 (11 paired resection samples from inflamed and uninflamed ileum of CD patients), all the stromal subsets, including *CXCL12*^+^, *CD90*^+^, *CD55*^+^, *PDPN*^+^, and *CD73*^+^ cells, were found to show a higher abundance and expression in inflamed tissue of CD patients compared to noninflamed samples (Supplementary Figure S4A-C). These data illustrate that the distribution of stromal subsets differs between the 2 types and intestinal locations of human IBD.

### IBD Therapies Are Partly Capable of Restoring Stromal Subset Abundance

**Figure 4.**
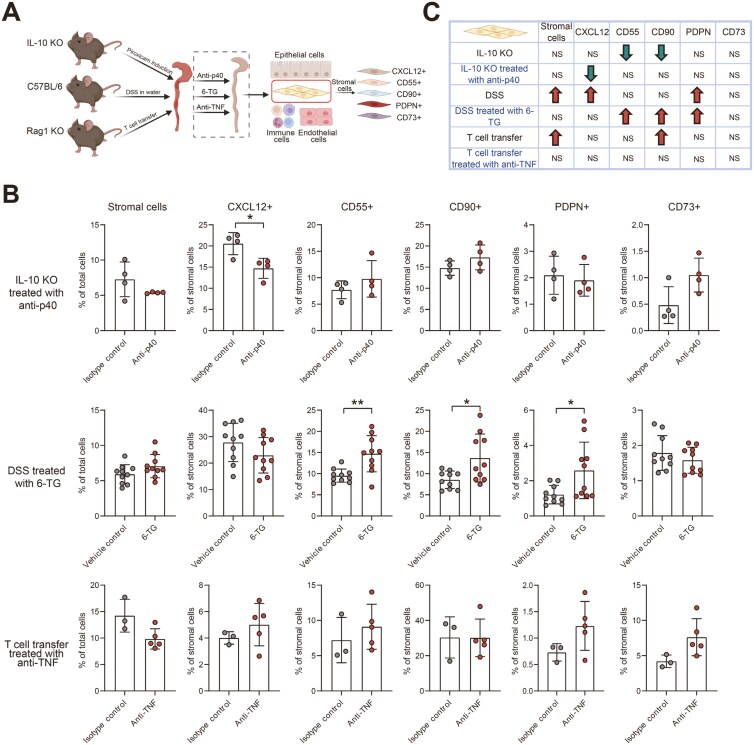
Effect of inflammatory bowel disease (IBD) therapies on stromal subset abundance. A, Schematic overview of the experiment set-up, 3 different experimental colitis models were used. Commonly used IBD therapies, including anti-TNF, 6-TG, and anti-p40 were applied to the mice in the different models. Epithelial cells, immune cells, and endothelial cells were excluded. Stromal cell subsets were identified by stromal markers. B, Bar graph of the percentage of stromal cells in 3 colitis mouse models. In IL-10 knockout colitis treated with anti-p40, control, *n* = 4, anti-p40, *n* = 4; in dextran sulfate sodium-induced colitis treated with 6-TG, control, *n* = 10, 6-TG, *n* = 10; in T-cell transfer colitis treated with anti-TNF, control, *n* = 3, anti-TNF, *n* = 5. C, Overview of the trend of percentage of stromal subsets in 3 colitis mouse models after individual treatment. The direction of the arrows indicate a significant increase or decrease (*P* ≤ 0.05). The data are presented as the mean ± SD. Non-paired two-tailed *t*-test or Mann–Whitney *U* test was performed. *P ≤ 0.05, **P ≤ 0.01. Abbreviation: NS, no significance.

To investigate whether commonly used IBD therapies could affect stromal subset abundance, anti-p40, 6-TG, and anti-TNF treatments were given to IL-10 KO, DSS, and T-cell transfer–induced colitis mice, respectively. All these therapies alleviated the induced colitis in mice; in the treatment groups compared to vehicle or isotype control groups the MEICS score and the relative colon weight were decreased (Supplementary Figures S5-S7B and C) and histological analysis showed less immune cell infiltration (Supplementary Figures S5-S7D). Next, flow cytometry analysis was performed to evaluate the stromal subset composition post-treatment (Figure 4A). Interestingly, the increase in the total amount of stromal cells during colitis was reverted after the treatment with anti-TNF in the T-cell transfer model, although this did not reach statistical significance (Figure 4B). As for the stromal subsets, the abundance of CXCL12^+^ stromal cells decreased upon anti-p40 treatment in the IL-10 KO colitis mice (*P* = 0.02, Figure 4B and C). In DSS-induced colitis, an increased number of CD55^+^, CD90^+^, and PDPN^+^ stromal cells was present after treatment with 6-TG (Figure 4B and C). Remarkably, the percentage of PDPN^+^ stromal cells was already higher during colitis. In the T-cell transfer for colitis, there was a trend, although not significant, toward an increase in the abundance of PDPN^+^ and CD73^+^ stromal cells (*P* = 0.13 and *P* = 0.08, respectively) after treatment with anti-TNF (Figure 4B and C). Taken together, these data indicate that IBD therapies are actively inducing stromal subset changes, but do not completely normalize the stromal component.

Next, we wanted to evaluate whether these therapies directly affect stromal subset composition. Therefore, fibroblasts from C57/Bl6 mice (representative of the DSS and T-cell transfer models) were treated with 6-TG and anti-TNF. In addition, fibroblasts were isolated from IL-10 KO mice and treated with anti-p40. After treatment, stromal subset marker expression was analyzed. The percentage of stromal cells was not affected by anti-p40 therapy (Supplementary Figure S8A). C57/BL6-derived fibroblasts treated with 6-TG showed an increased abundance of CXCL12^+^, and a slightly increased abundance of PDPN^+^ (Supplementary Figure S8B). Treatment with anti-TNF did not change any of the subset markers (Supplementary Figure S8C). These results indicate that only 6-TG could directly affect stromal subset marker expression *in vitro*, which was also reflected *in vivo* for PDPN expression.

### Knockdown of CXCL12 and CD55 Decreases the Proliferation and Migration of Murine Fibroblasts

To further investigate the function of the stromal subsets, we first evaluated whether stromal cells, and more specifically fibroblasts, isolated from an inflammatory milieu maintain their phenotype and specific marker expression *in vitro*. Colonic fibroblasts from DSS-induced and IL-10 KO colitis mice were isolated, cultured, and used between passages 5-10. Unfortunately, no viable fibroblast cultures could be established from the T-cell transfer mice. In contrast to our *in vivo* observations, almost all cultured intestinal fibroblasts derived from healthy and colitis mice showed high CD90^+^ and CXCL12^+^ expression *in vitro* (Supplementary Figure S9A and B). Also, differences in PDPN expression upon DSS-induced colitis were lost upon cell culture (Supplementary Figure S9A, B), while CD73 was not detected in *in vitro* fibroblasts (data not shown). These data show that the specific, colitis-associated characteristics of the fibroblasts cannot be maintained *in vitro* in regular cell cultures.

**Figure 5.**
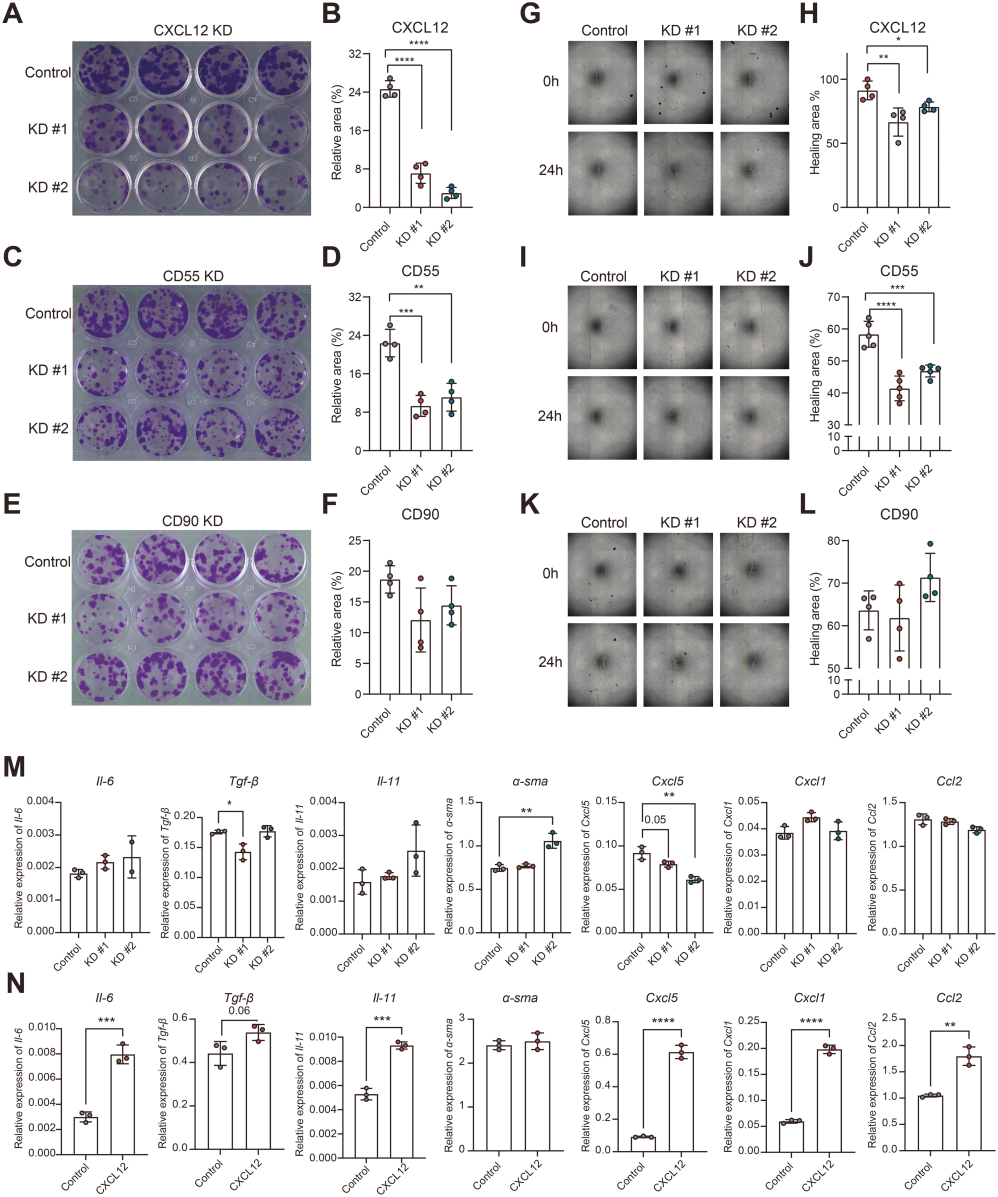
Knockdown of CXCL12 and CD55 decreases the proliferation and migration of murine fibroblasts. Knockdown of CXCL12, CD55, and CD90 in murine fibroblasts 3T3-J2 was stably established. Representative image from the colony formation assay, showing strongly decreased proliferation after KD of A, CXCL12, C, CD55, and no difference after KD of E, CD90. B, D, and F, Bar graph represented the statistical results of the relative colony area (*n* = 4 technical replicates). Representative phase-contrast microscope images showing the area covered by the cells at 0 and 24 hours after wounding, showing decreased wound healing after KD of G, CXCL12, I, CD55, and no difference after KD of K, CD90. H, J, and L, Quantification of the wound healing assay. The data were normalized to the wound width of each group at 0 hours (*n* = 4–5 technical replicates). M, Quantitative polymerase chain reaction (qPCR) analysis of 3T3 cells after KD of CXCL12. Bar graph of relative expression of *Il-6*, *Tgf-β*, *Il-11*, *α-sma*, *Cxcl5*, *Cxcl1*, and *Ccl2*. N, 3T3 cells were incubated with 50 ng/mL recombinant CXCL12 protein for 6 hours and qPCR analysis was performed. Bar graph of relative expression of the same genes. All experiments were repeated 3-4 times independently. Nonpaired two-tailed *t*-test was performed. *P ≤ 0.05, **P ≤ 0.01, ***P ≤ 0.001, and ****P ≤ 0.0001.

Given the fact that fibroblasts do not maintain their full phenotype in culture and since we wanted to know the specific role of some markers, we generated fibroblasts in which the subset markers CXCL12, CD90, and CD55 were knocked down using shRNA. The KD efficiency was validated via qPCR (Supplementary Figure S10A) and flow cytometry analysis (Supplementary Figure S10B), showing markedly decreased expression compared to nontargeting controls. To investigate their role in the proliferation and migration of fibroblasts, colony formation, and wound healing assays were performed, respectively (Supplementary Figure S11). Our data showed that the KD of both CXCL12 and CD55 significantly decreased the proliferation of 3T3 murine fibroblasts (Figure 5A-D), whereas CD90 KD did not change the proliferation rate significantly (Figure 5E and F). Consistently, the migration capacity of 3T3 murine fibroblasts was significantly decreased after the KD of CXCL12 and CD55, but not for CD90 (Figure 5G-L). These data highlight the important role of CD55 and CXCL12 in fibroblast proliferation and migration.

To further elucidate the involvement of CXCL12 on fibroblast behavior, we conducted qPCR analysis on 3T3 fibroblasts with shRNA-mediated CXCL12 KD. Interestingly, we observed that the expression levels of key markers associated with activated fibroblasts, such as *Il-6*, *Tgf-β*, *Il-11*, *α-sma*, *Cxcl5*, *Cxcl1*, and *Ccl2*, remained unchanged following CXCL12 KD, except for *Cxcl5*, which was downregulated upon CXCL12 KD (Figure 5M). Noteworthy, the addition of exogenous recombinant CXCL12 protein to murine fibroblasts led to an elevation in the expression levels of all these markers, except for *α-sma* (Figure 5N). These findings suggest that CXCL12 could regulate fibroblasts in an autocrine manner.

### Knockdown of CXCL12 in Fibroblasts Reduces Epithelial Migration

**Figure 6.**
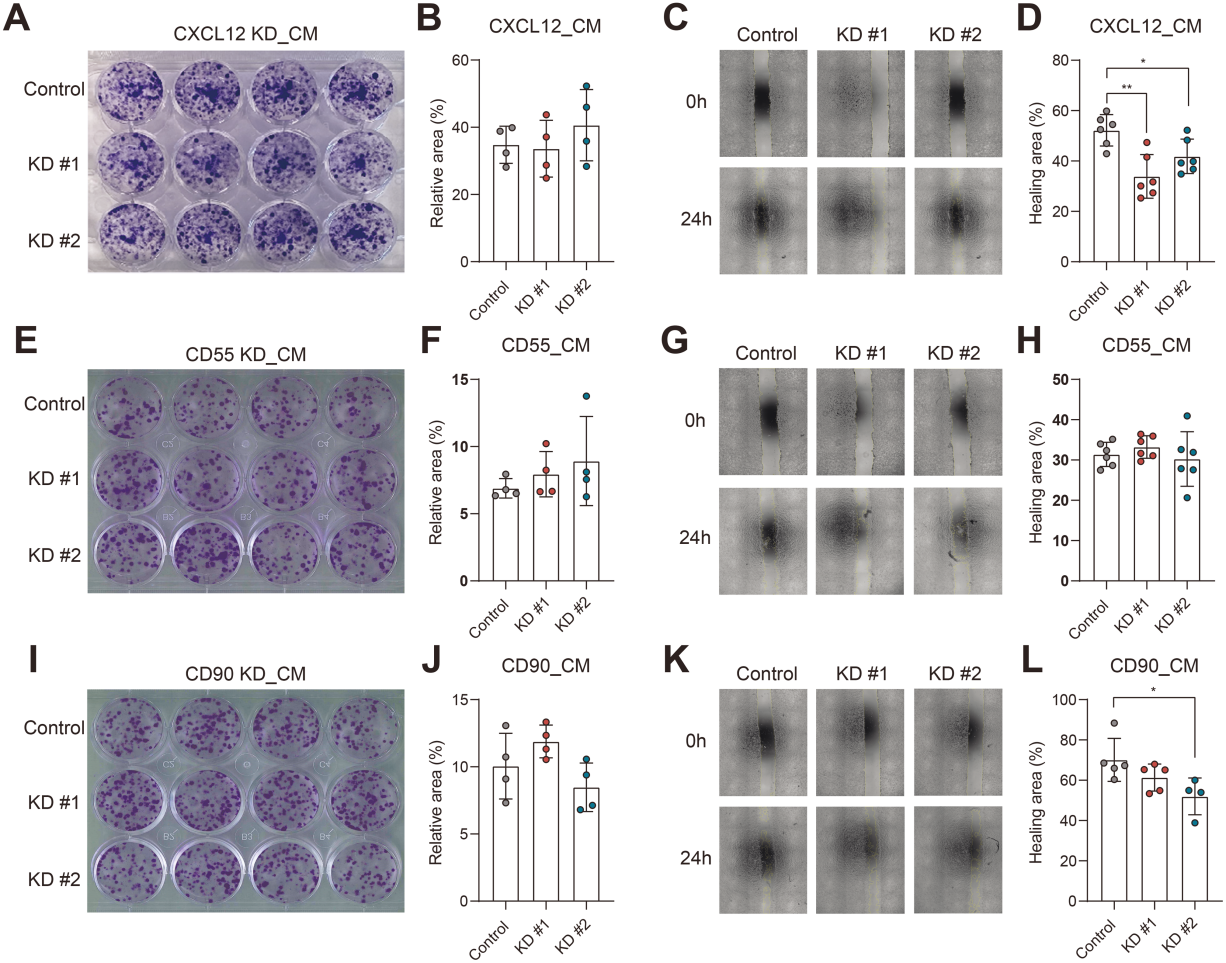
Knockdown-CXCL12 medium from murine fibroblasts decreases the wound-healing capacity of murine epithelial cells. Epithelial cells CT26 were incubated with the knockdown (KD)-conditioned medium (CM) from murine fibroblasts 3T3-J2. Representative image from the colony formation assay of CT26, showing no difference after incubation with A, KD-CXCL12 E, k KD-CD55, and I, KD-CD90 CM. B, F, and J, Bar graph represented the statistical results of the relative colony area (*n* = 4 technical replicates). Representative phase-contrast microscope images showing the area covered by the cells at 0 and 24 hours after wounding, showing decreased wound healing after incubation with C, KD-CXCL12-CM and G, no difference after KD-CD55, and K, KD-CD90 CM. (D, H, and L) Quantification of the wound-healing assay. The data were normalized to the wound width of each group at 0 hours (*n* = 4–6 technical replicates). All experiments were repeated 3-4 times independently. Nonpaired two-tailed *t*-test was performed. *P ≤ 0.05, **P ≤ 0.01.

Next, we aimed to investigate the paracrine effects of fibroblasts on epithelial cells. To investigate whether epithelial proliferation and migration were affected, CM from 3T3 fibroblasts with nontargeting control, CD90, CD55, or CXCL12 KD constructs was collected. Murine epithelial CT26 cells were exposed to CM and proliferation was assessed. Our data showed that the proliferation of CT26 cells was not affected when stimulated with CXCL12 KD fibroblast CM (Figure 6A and B). In contrast, the migration of CT26 cells was decreased upon stimulation with CXCL12 KD fibroblast CM medium, compared to a nontargeting control (Figure 6C and D). This indicates that the KD of CXCL12 in fibroblasts can reduce their migration. CD55 and CD90 KD fibroblast CM did not show any significant effect on the proliferation and migration of CT26 compared to a nontargeting control (Figure 6E-L). In conclusion, in contrast to CD90 and CD55 expression, CXCL12 expression on fibroblasts seems to affect epithelial cell migration.

### Fibroblast KD of CXCL12 and CD55 Influences T-Cell Activation

**Figure 7.**
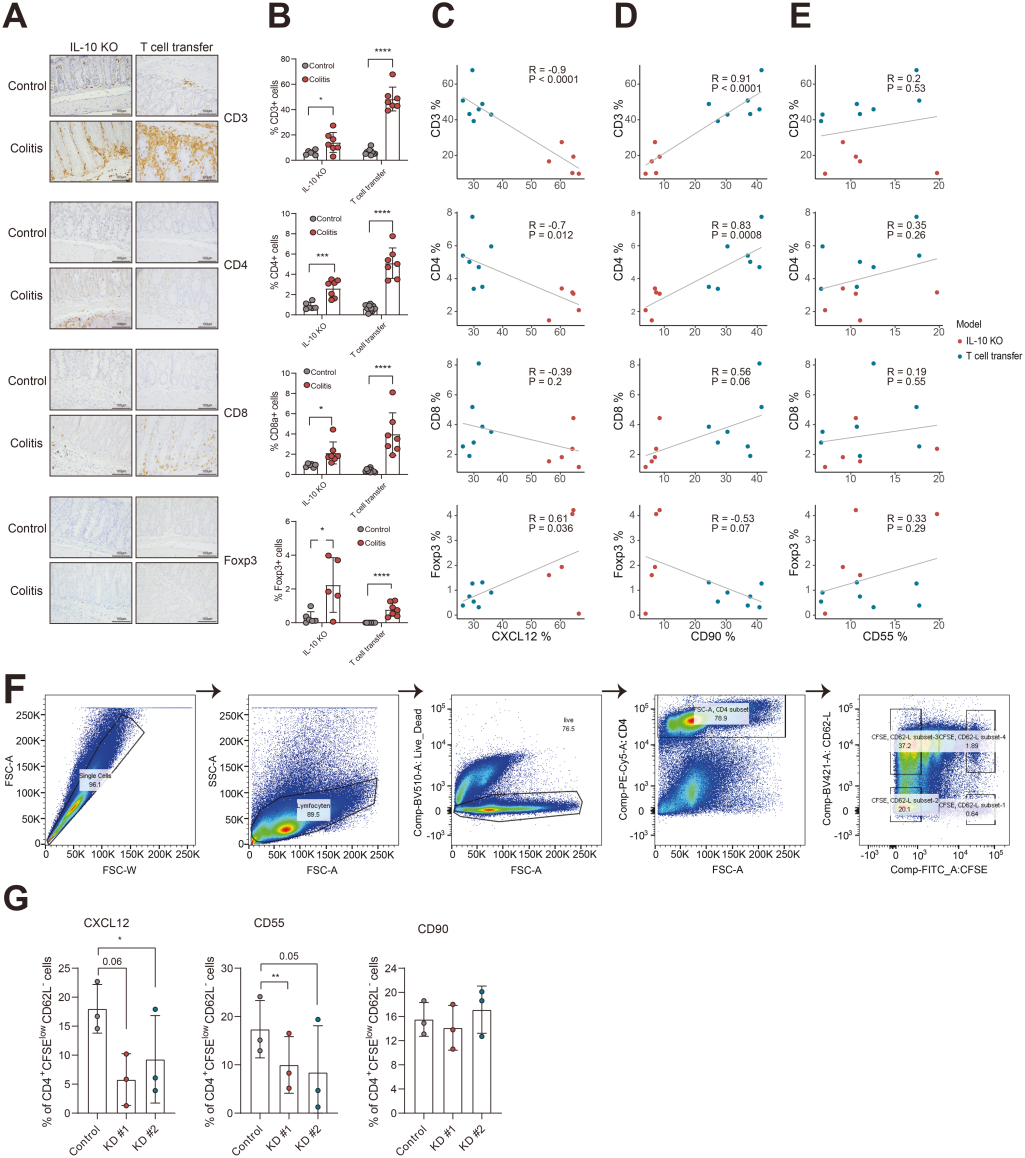
CXCL12 have a significant chemoattractive effect on T cells in inflammatory bowel disease (IBD) mouse models. A, Representative immunohistochemical staining of CD3, CD4, CD8, and Foxp3 in IL-knockout colitis and T-cell transfer colitis models. Scale bar: 100 μm. B, Quantification analysis based on the positively stained area in random views. Nonpaired two-tailed *t*-test was performed to assess a statistical significance between control and colitis groups. *P ≤ 0.05, ***P ≤ 0.001, ****P ≤ 0.0001. Pearson R correlation of percentage of C, CXCL12^+^ stromal cells (flow cytometry data obtained from *in vivo* experiment) D, CD90^+^ stromal cells, and E, CD55^+^ stromal cells and the percentage of T-cell infiltrate found in inflamed colonic tissue of IBD mouse models. F, Gate strategy of the flow cytometry of T cells which were cocultured with murine fibroblasts 3T3-J2 knocked down of CXCL12, CD55, and CD90. CD4^+^ T cells were selected and CD4^+^CFSE^low^CD62L^−^ cell population was regarded as the most activated T cells. G, Bar graph of the percentage of activated T cells after cocultured with KD-CXCL12, KD-CD55, and KD-CD90 fibroblasts (*n* = 3 independent experiments). Paired two-tailed *t*-test was performed. *P ≤ 0.05, **P ≤ 0.01.

Since T cells play a pivotal role in the immune response underlying IBD pathogenesis,^37^ we wanted to investigate whether stromal subsets are associated with a change in the abundance of T cells in IBD mouse models. Immunohistochemical staining indicates an increase in the total amount of T cells (CD3^+^) and CD4^+^, CD8^+^, and Foxp3^+^ T-cell subsets in colitis compared to control groups (Figure 7A and B). Next, the abundance of CXCL12^+^, CD90^+^, and CD55^+^ stromal cell subsets was correlated with the number of infiltrating T cells. Interestingly, the abundance of CXCL12^+^ stromal cells showed a negative correlation with the total percentage of T cells and more specifically with CD4^+^ T cells (Figure 7C). A positive correlation between the percentage of CXCL12^+^ stromal cells and the percentage of Foxp3 T-regulatory cells was observed (Figure 7C). On the contrary, the abundance of CD90^+^ stromal cells was positively correlated with total T-cell and CD4^+^ T-cell count while negatively correlated with Foxp3 T cells, although not significant (*P* = 0.07, Figure 7D). The correlation of the percentage of CD55^+^ stromal subsets with T cells showed no significant changes (Figure 7E). These data show that a correlation exists between stromal cell subsets and various T-cell subsets between the different murine IBD models.

To explore whether fibroblast subsets could directly affect CD4^+^ T-cell proliferation, fibroblasts with a specific subset marker deletion were cocultured with OT-II splenocytes (OVA-specific CD4 T cells^38^). Following a 3-day coculture with indicated fibroblasts, CD4^+^ T cells were collected for flow cyctometry analysis, using the gating strategy depicted in Figure 7F. The CD4^+^CFSE^low^CD62L^−^ cell population was identified as the most activated T-cell population.^39,40^ Coculture of CD4^+^ T cells with CXCL12 KD fibroblasts resulted in fewer activated T cells (Figure 7G). This was also observed after coculture with CD55 KD fibroblasts, although this did not reach statistical significance (Figure 7G). The presence or absence of CD90 expression on fibroblast did not affect the percentage of activated T cells (Figure 7G). These data reveal that especially CXCL12 affects CD4 T cell activation *in vitro*, which is in line with our previous observation *in vivo*.

## Discussion

The current study shows that the abundance of stromal cell subsets is altered upon induction of colitis and differs substantially among 3 established colitis mouse models. Interestingly, the spatial localization of CD90^+^ stromal cells seems to change during colitis. Moreover, our data show that commonly used IBD therapies can change the stromal subset composition but cannot completely restore the “normal” stromal subset abundance. Interestingly, the expansion of the total stromal cell population was only reverted by anti-TNF-α therapy. *In vitro* experiments illustrated that CXCL12^+^ stromal cells might play a central role in IBD pathogenesis since they affect epithelial cell migration and coculture of CXCL12 KD stromal cells with T cells resulting in less activated T cells.

For a long time, fibroblasts have been considered a homogenous cell population which provided tissue structure and production of extracellular matrix (ECM) components.^41^ However, more recent work identified fibroblast subsets with distinct functions.^12,13,42^ The increasing knowledge and data make it challenging to explore their role in IBD pathogenesis since the heterogeneity of fibroblasts and the lack of clear definitions of fibroblast subsets. Landmark papers on fibroblast subsets in IBD patients describe at least 4-5 fibroblast subsets.^13,30^ To date, these subsets have not been studied in the most commonly used mouse models of IBD. Our current data show that the stromal subsets in the colon display model-dependent changes during colitis. A comparison of stromal subsets in 3 colitis models of IBD revealed that none of the experimental colitis models closely mimics human IBD. The DSS model is always thought to represent UC, but our data showed, for example, the opposite response in CXCL12 stromal subset upon colitis induction in mice and humans, indicating the importance of keeping this in mind when analyzing the function of stromal subsets in IBD pathogenesis. Furthermore, the abundance of stromal subtypes could dynamically change with the progression of the disease, from initiation to sustained and chronic colitis, which we did not study in detail in this work, but was briefly touched upon in Supplementary Figure S3. Recently, 3 major lineages of fibroblast subsets were identified in the colon of chronic DSS-induced colitis using single-cell RNA sequencing analysis. The authors defined fibroblast 1 as CD55^+^Grem1^+^Cd81^−^Procr^−^ and showed that this subset can support the epithelial stem cell niche by highly expressing Grem1. Fibroblast subset 2 was α-SMA^+^ and showed the absence of CD55 and CD81 and participated in the regulation of the bone morphogenetic proteins pathway. Finally, fibroblast subset 3 was defined as CD55^+^CD81^+^Pcolce2^+^C3^+^Procr^−^ and played a role in the remodeling of the intestinal ECM.^43^ These data are a valuable addition to our acute DSS experiments. We could infer that stromal subsets vary between the acute, chronic, and recovery phases of IBD. This also indicates the difficulty of the field of fibroblast research since the establishment of fibroblast subsets is still under debate and not firmly established.

One of the stromal subsets showing major differences is the CXCL12 expressing subset. As a strong chemotactic for lymphocytes, CXCL12 is important in embryogenesis, hematopoiesis, angiogenesis, intestinal poly formation, and inflammation.^14,15^ This subset was increased in DSS-induced colitis and downregulated after treatment with anti-p40 in IL-10 KO colitis. Our *in vitro* experiments showed that CXCL12 KD fibroblasts proliferated less and showed decreased migratory capacities. Interestingly, exogenous recombinant CXCL12 led to activation of fibroblasts by upregulation of *IL-6* and *IL-11* among others, which indicates CXCL12 could regulate fibroblasts through autocrine signaling. Interestingly, previous research has shown increased expression of CXCL12 in intestinal epithelial cells in IBD patients.^44^ These epithelial cells could also serve as an additional potential source of CXCL12 that influences fibroblast behavior and function. Furthermore, we also found that CXCL12 KD fibroblasts stimulated the migration of epithelial cells in a paracrine manner. It was reported before that fibroblast-derived CXCL12 is upregulated in the inflamed murine colon, which results in the recruitment of lymphocytes toward the colonic mucosa. Interestingly, this seems to be regulated by epithelium-derived Indian Hedgehog signals, showing the complex interplay among epithelial cells, immune cells, and fibroblasts in intestinal homeostasis and inflammation.^45^ The interaction between fibroblasts and T cells in a CXCL12-dependent manner was also previously shown in the oncology field. CXCL12-expressing cancer-associated fibroblasts attract T cells and promote them to differentiate into Foxp3^+^ T cells, thereby generating an immunosuppressive environment.^46,47^ Interestingly, our current data showed that coculture of CD4^+^ T cells with CXCL12 KD fibroblasts resulted in fewer activated T cells, possibly indicating that CXCL12-negative stromal cells have immunosuppressive effects in the process of colitis.

Current therapies for IBD are focused on alleviating (inflammatory) symptoms. Anti-TNF therapies block the TNF-mediated activation of the proinflammatory pathways and inhibit the classic hallmarks of chronic inflammation. Next to the direct effects on immune cells, little is known about the effects of anti-TNF therapy on stromal subsets. We show that the colitis-induced increase in stromal cells is reduced after the anti-TNF therapy, although not reaching statistical significance. This was accompanied by an increase in PDPN^+^ and CD73^+^ stromal cells. It is unknown whether these are direct or indirect effects of anti-TNF therapy on stromal cells. At least, our *in vitro* data showed no changes in these markers after treatment of colon-derived murine fibroblasts with anti-TNF. Previously, it was reported that anti-TNF resistance is associated with specific stromal subset abundance. Interestingly, this stromal subset showed also high PDPN expression.^30^ Therefore, studying the function of the stromal subset may offer novel insights into the efficacy or optimization of anti-TNF therapy in the treatment of IBD.

P40 is the sharing subunit of IL-12 and IL-23. Interleukin-12 provides a Th1 and IL-23 provides a Th17 response and consequently, T cells start producing proinflammatory cytokines like TNF-α and IFN-γ.^48,49^ It is reported that stromal cells can influence the secretion of IL-23/IL-12.^50,51^ Our data show that anti-p40 therapy (clinically available as ustekinumab) is capable of reducing the amount of CXCL12^+^ stromal cells *in vivo*, potentially limiting intestinal inflammation. Previously, it was also reported that blockade of the CXCL12/CXCR4 axis using a CXCR4 antagonist could ameliorate DSS-induced colitis.^52^ In addition, anti-p40 therapy is found to partially restore the colitis-induced decreased percentage of CD90^+^ stromal subset (*P* = 0.19). CD90^+^ colonic (myo)fibroblasts express programmed death-ligand 1 (PD-L1), which contributes to the suppression of Th1 response in UC patients.^21^ Taken together these data indicated a potentially important role for CD90 expressing fibroblasts in regulating immune cells function during colitis.

In conclusion, we show that the stromal subset abundance differs among 3 established colitis mouse models, possibly reflecting the diversity in human IBD. The CXCL12^+^ stromal subset, which was increased upon DSS-colitis induction, might play an important role in IBD pathogenesis since it affects both epithelial and immune cells. Although changes in stromal abundance were observed, treatment with anti-p40, anti-TNF, or 6-TG therapy could not restore the original stromal subset composition. Overall, our results provide important new insights into the role of stromal cell subsets in IBD pathogenesis and provide a stepping stone to further unravel their exact role and study new therapeutic strategies for IBD patients.

## Supplementary Data

Supplementary data is available at *Inflammatory Bowel Diseases* online.

izae255_suppl_Supplementary_Material

## Data Availability

The authors confirm that the data supporting the findings of this study are available within the article [and/or its supplementary materials].
