# Supplementary material for: Stromal Cell Subsets Show Model-Dependent Changes in Experimental Colitis and Affect Epithelial Tissue Repair and Immune Cell Activation
Source: Inflamm Bowel Dis. 2025 Mar 18;31(4):1051–66. doi: 10.1093/ibd/izae255 (PMC11985400; doi:10.1093/ibd/izae255)
Supplement: izae255_suppl_Supplementary_Material [file izae255_suppl_supplementary_material.pdf]

Figure S1. Gate strategy of the flow cytometry analysis. A: Dead cells, Epcam^{+}, CD45^{+} cells, and CD31^{+} cells were excluded. **B:** Representative gating for stromal subsets.

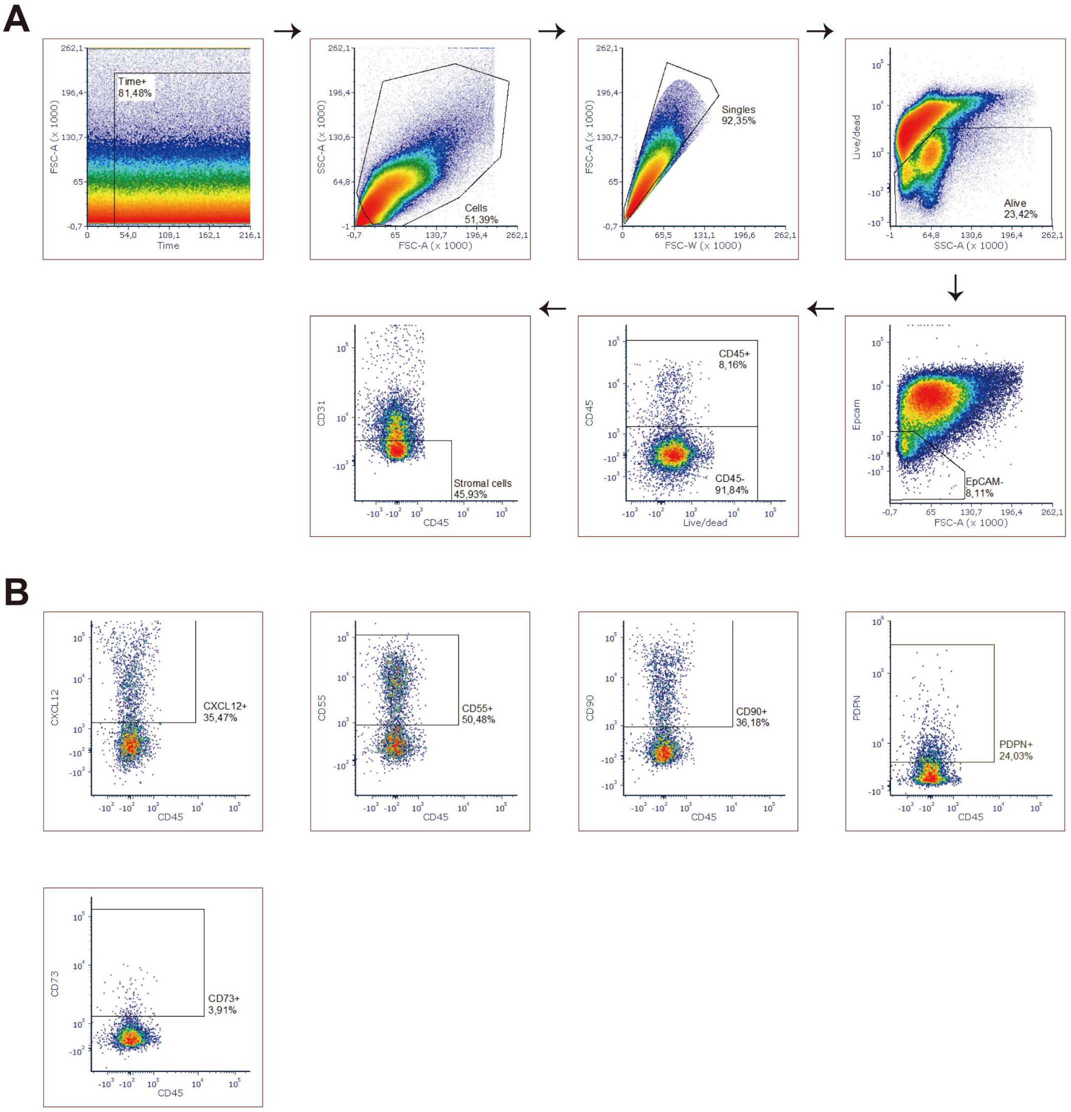

Figure S2. Experimental colitis mouse models were successfully induced. A: Body weight of mice was measured and expressed as the percentage of body weight on day 0 in IL-10 KO colitis, DSS-induced colitis and T cell transfer colitis. **B:** MEICS score of the mice on sacrifice day in three colitis mouse models. **C:** Hematoxylin and eosin staining (scale bar: 50μm) of the colon in three colitis mouse models. **D:** Percentage of CD45^{+} cells in the colon in three colitis mouse models. Non-paired two-tailed t-test was performed. *p≤0.05, ***p≤0.001, ****p≤0.0001.

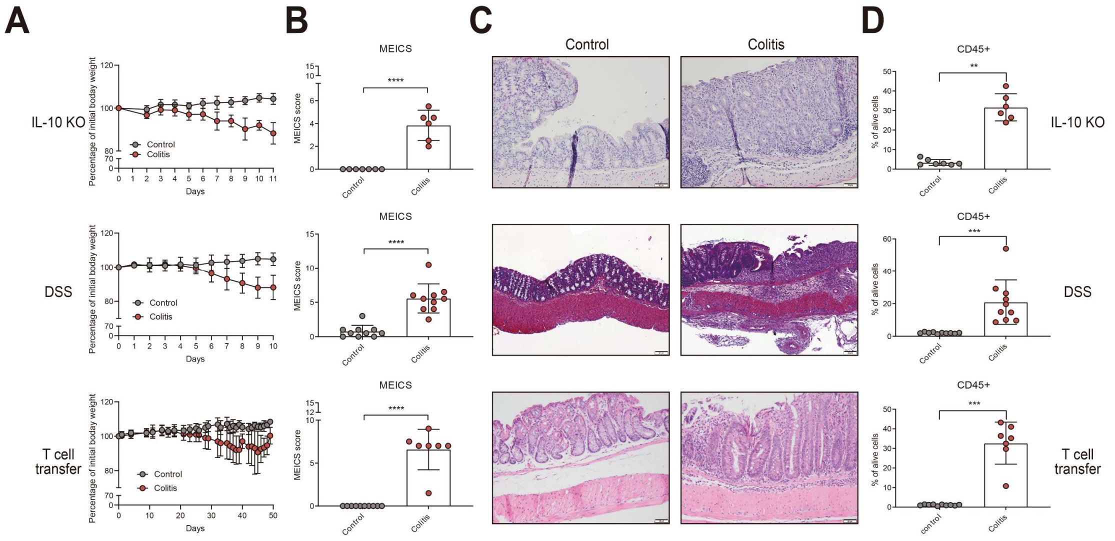

Figure S3. Composition of stromal subsets abundance in DSS-induced colitis mouse. UMAP plot of stromal cells in GSE48794. *Cxcl12* **(A)**, *Cd55* **(B)**, *Cd90* **(C)**, *Pdpn* **(D)**, *and Cd73* **(E)** positive stromal cells were indicated in purple and the negative stromal cells were grey. Stromal cluster was identified with the expression of *Col1a1*, *Pdgfra*, *and Spon2.* **(F)** The percentage of positive stromal cells at different time points.

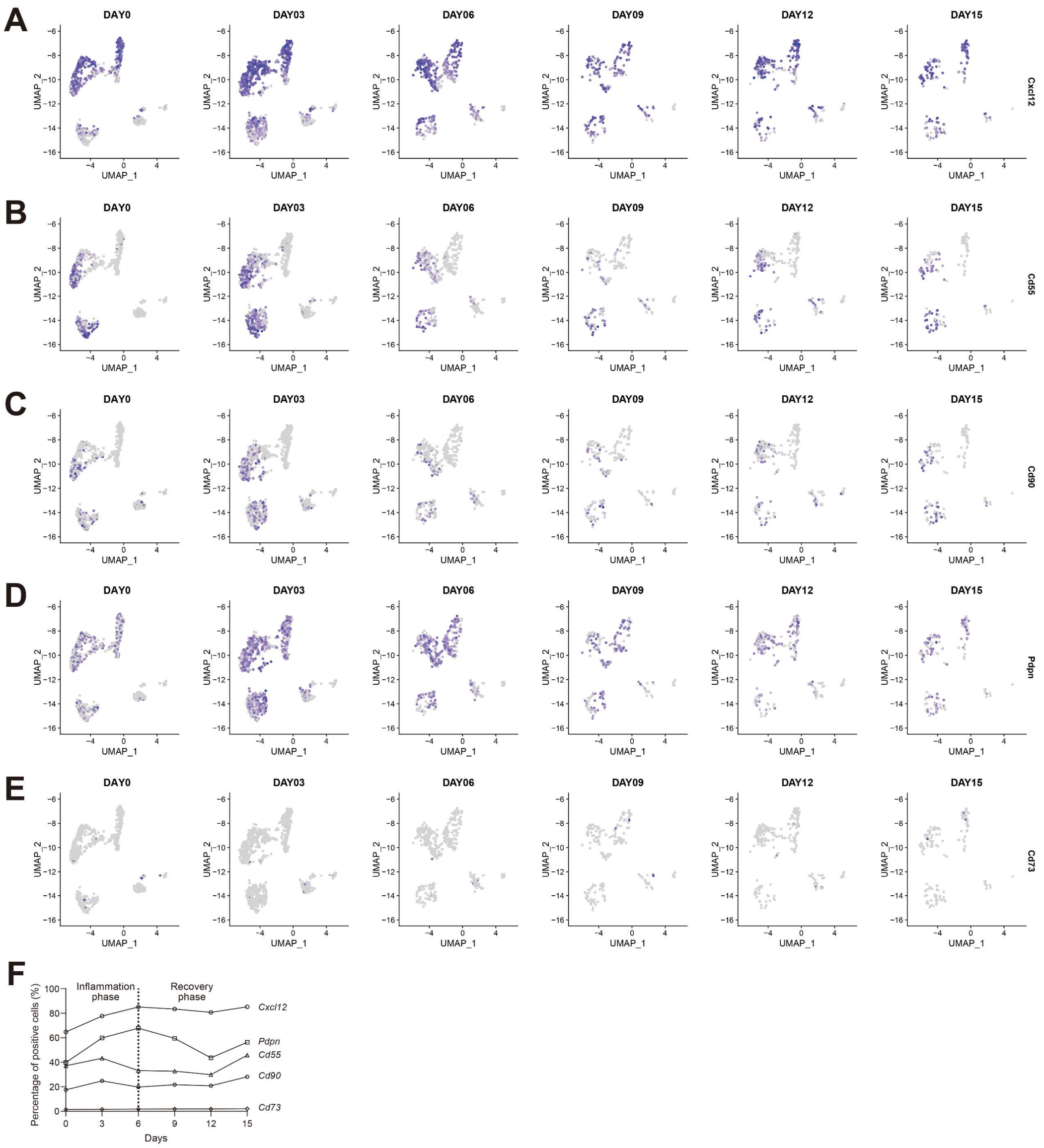

Figure S4. Composition of stromal subsets abundance in human iCD stromal cells. A: UMAP plot of stromal cells in GSE134809. *CXCL12*, *CD55*, *CD90*, *PDPN*, and *CD73* positive stromal cells were indicated in purple in non-inflamed (left) and inflamed (right) ileum from CD patients. Markers for fibroblasts (*CCL13*, *CCL8*) and activated fibroblasts (*CXCL8*, *CXCL3*, *CXCL1*, *and CXCL6*) were used to identify stromal cells. **B:** Rate of positive and negative stromal cells Fisher's exact test was performed to assess statistical significance. **C:** Violin plots of expression of *CXCL12*, *CD55*, *CD90*, *PDPN* and *CD73* in stromal cells. Mann-Whitney test was performed. **p≤0.01, ***p≤0.001, ****p≤0.0001.

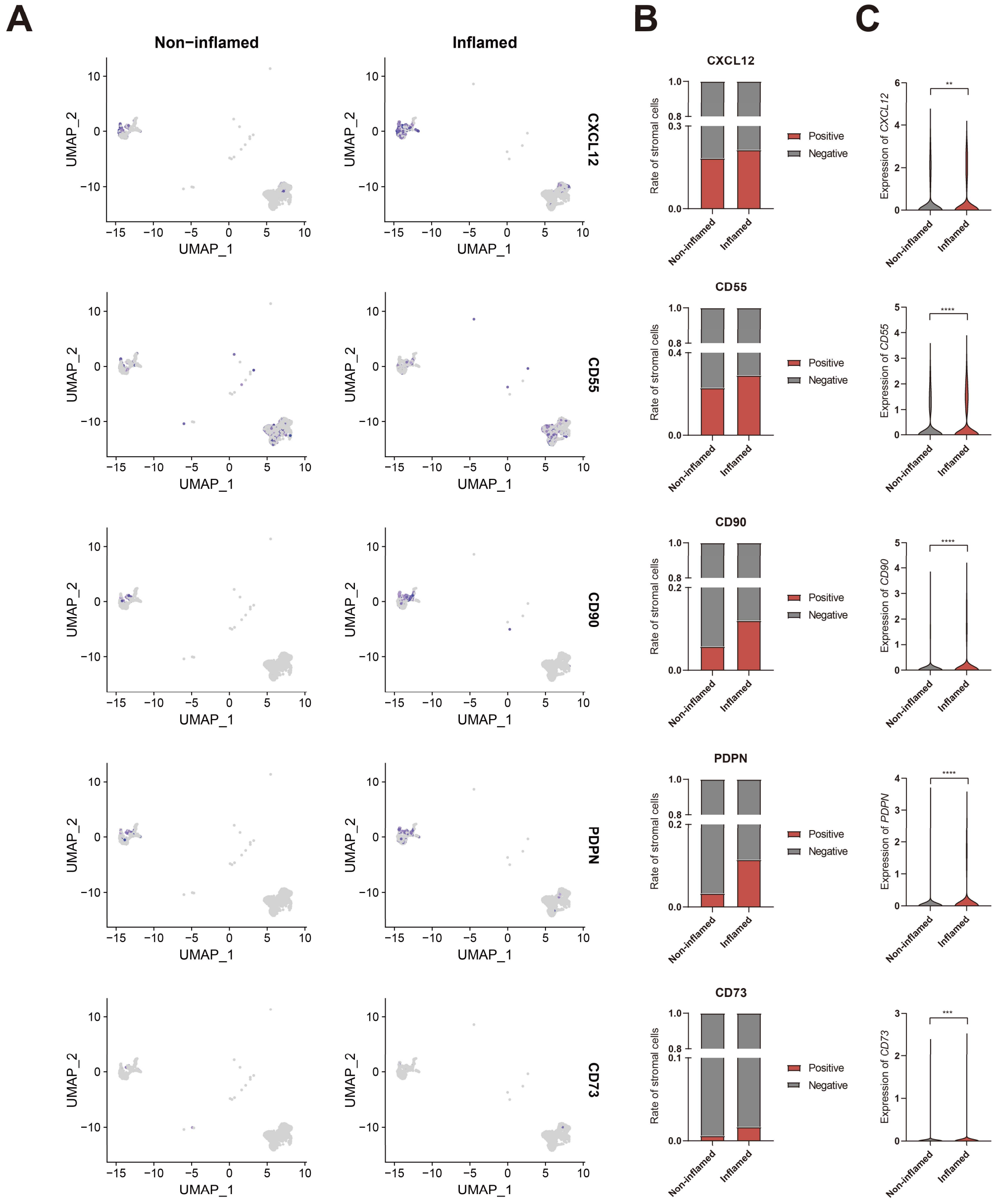

Figure S5. Anti-p40 treatment improved the inflammation in IL-10 KO colitis model. A: Body weight of mice expressed as the percentage of body weight at day 0. **B:** Colon weight/length ratio (g/cm) of the mice upon sacrifice. **C:** MEICS score of the mice in every group at day 9, -11, -14, -24 and -29 (sacrifice day). **D:** Representative hematoxylin and eosin staining of the colon. Scale bar: 200μm. Non-paired two-tailed t-test was performed to assess statistical significance.

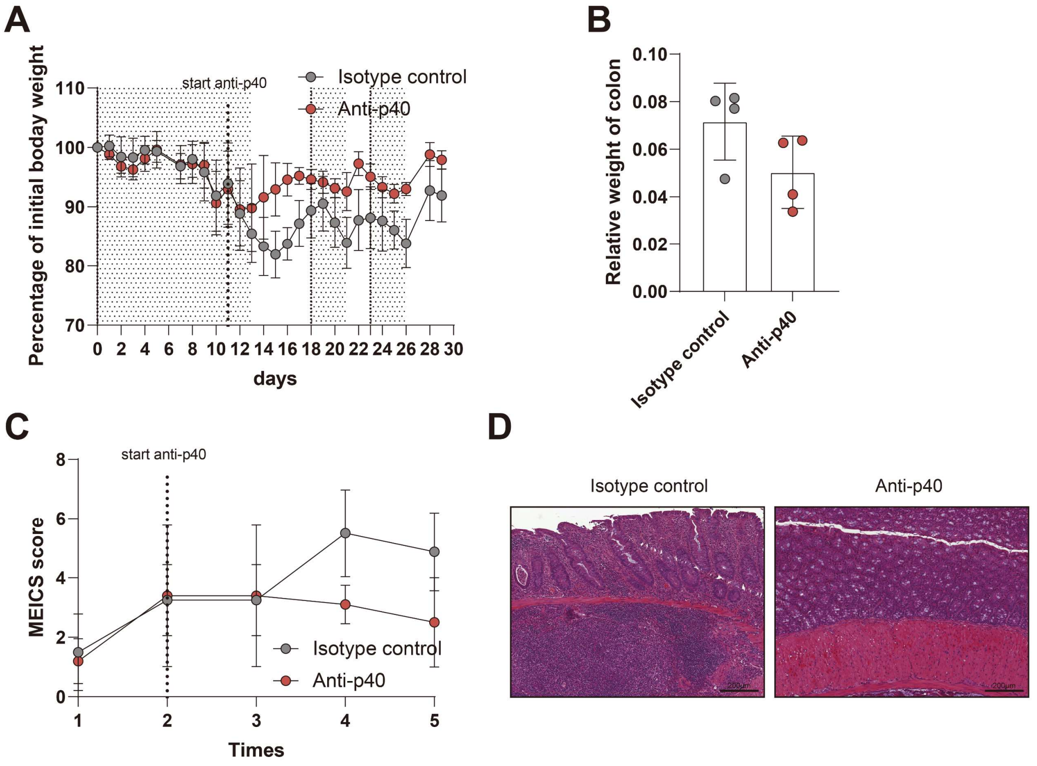

Figure S6. Anti-TNF treatment improved the inflammation in T cell tranasfer colitis model. A: Body weight of mice expressed as the percentage of body weight at day 0. **B:** Colon weight/length ratio (g/cm) of the mice upon sacrifice. **C:** MEICS score of the mice in every group at day 30, -37, -44 and -47 (sacrifice day). **D:** Representative hematoxylin and eosin staining of the colon. Scale bar: 200μm. Non-paired two-tailed t-test was performed to assess statistical significance. **p≤0.01.

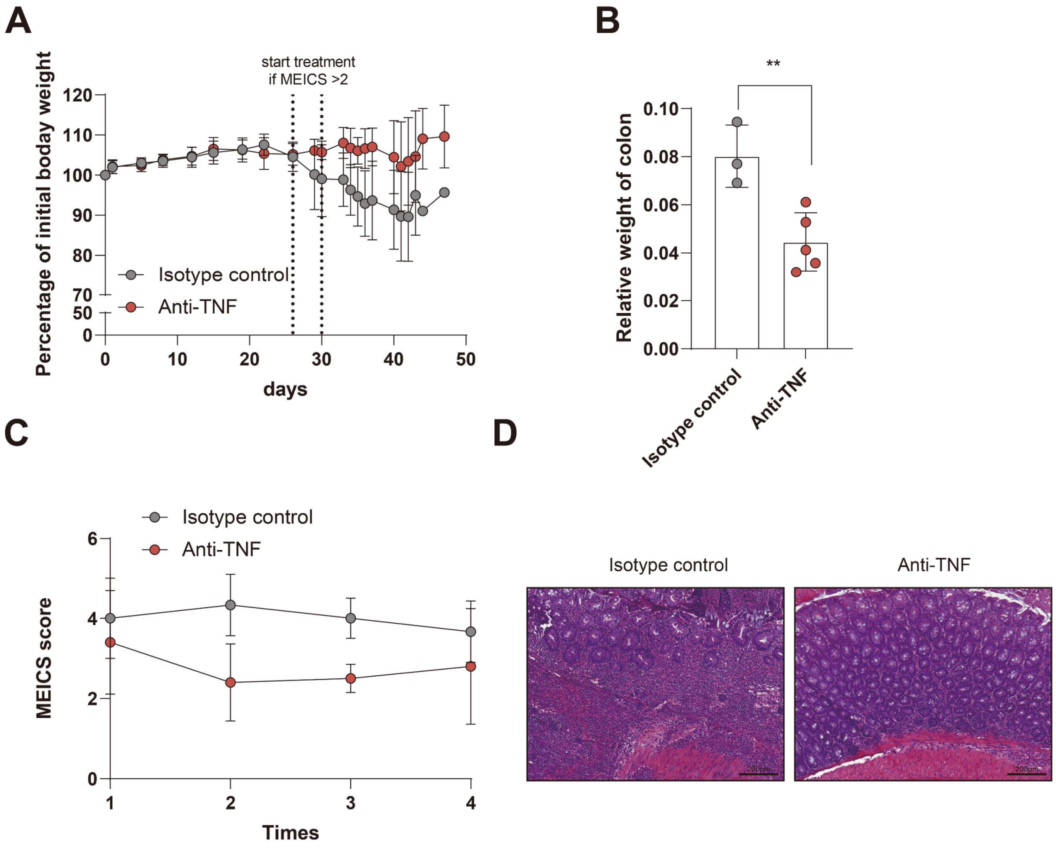

Figure S7. 6-TG treatment improved the inflammation in DSS-induced colitis model. A: Body weight of mice expressed as the percentage of body weight at day 0. **B:** Colon weight/length ratio (g/cm) of the mice upon sacrifice. **C:** MEICS score of the mice in every group at day 10 (sacrifice day). **D:** Representative hematoxylin and eosin staining of the colon. Scale bar: 200μm. Non-paired two-tailed t-test was performed to assess statistical significance. **p≤0.01.

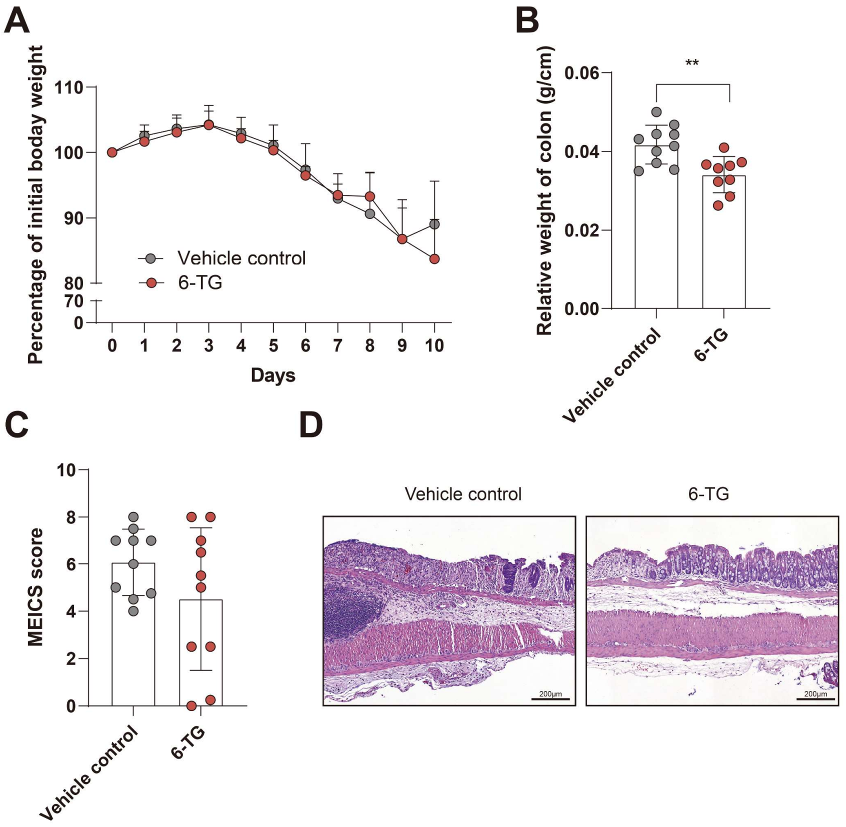

Figure S8. Effect of IBD therapies on murine primary fibroblasts. Primary fibroblasts were isolated from colon of IL-10 KO and C57BL/6 mice and treated with anti-p40, 6-TG and anti-TNF for 48 hours. Cells were then collected for flow cytometry analysis. Percentage of CXCL12, CD55, CD90, PDPN and CD73 on IL-10 KO originated fibroblasts treated with anti-p40 **(A)**, C57BL/6 originated fibroblasts treated with 6-TG **(B)** and C57BL/6 originated fibroblasts treated with anti-TNF **(C)**. n=3/4 independent fibroblasts. Non-paired two-tailed t-test was performed to assess statistical significance. *p≤0.05.

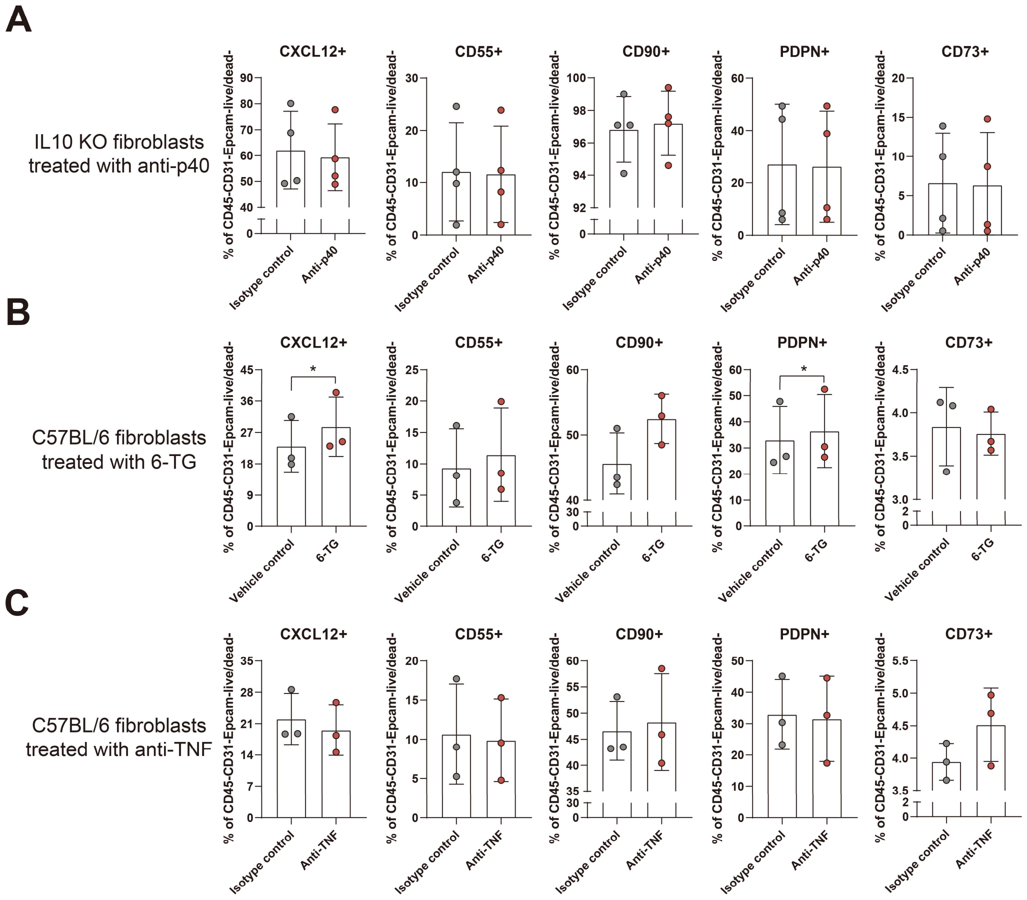

Figure S9 Colon-derived fibroblasts from experimental colitis mouse models lose the characteristics during the culturing. Dead cells, CD31$^+$ cells, CD45$^+$ cells, and Epcam$^+$ cells were excluded. **A:** Bar graph of the percentage of specific stromal cells in IL-KO mice colon derived primary stromal cells. **B:** Bar graph of the percentage of specific stromal cells in DSS-induced mice colon derived primary stromal cells. Non-paired two-tailed t-test was performed.

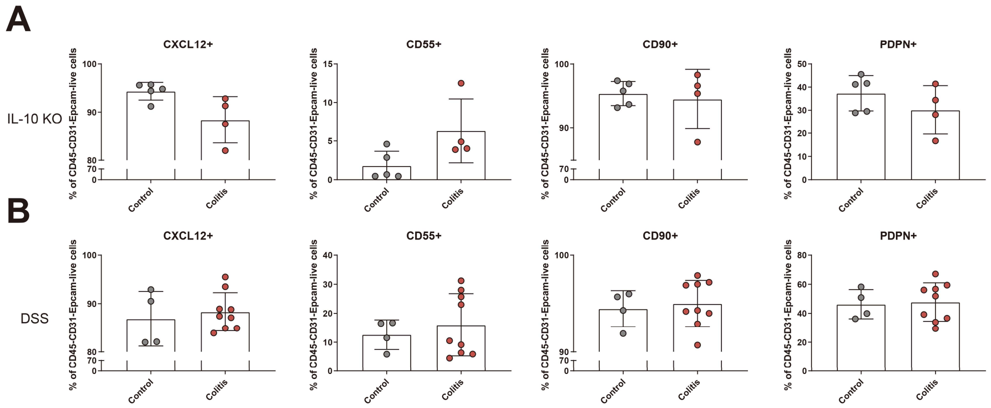

Figure S10. Knockdown efficiency of CXCL12, CD55 and CD90 in murine fibroblasts 3T3-J2. A: qPCR analysis of 3T3 cells after knockdown of CXCL12, CD55 and CD90. **B:** Flow cytometry analysis of 3T3 cells after knockdown of CXCL12, CD55, CD90. Grey: unstained; Red: control; Green and blue: KD #1 & KD #2.

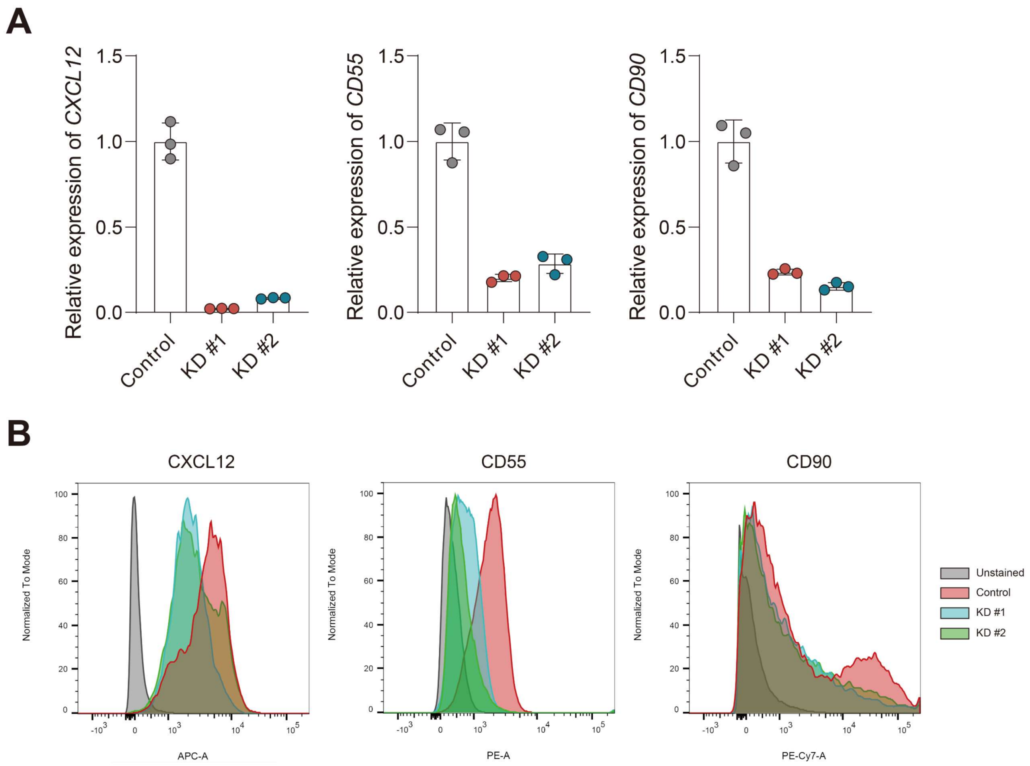

Figure S11. Schematic overview of the *in vitro* experiment set-up.

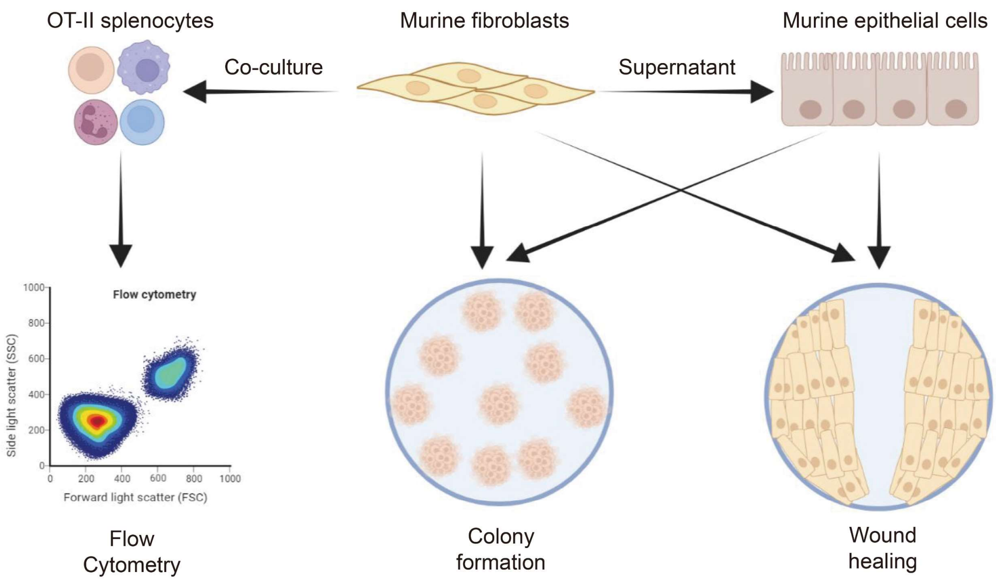

Supplementary Table 1: Antibodies for flow cytometry analysis

Antibodies	Fluorchrome	Dilution	Manufacturer
CD45.2	APC efluor 780	1:400	Invitrogen, eBioscience
CD31	Biotin	1:800	Invitrogen, eBioscience
Epcam	PerCP-Cyanine 5.5	1:800	Biolegend
CD90.2	PE Cyanine 7	1:400	Biolegend
CD73	V450	1:50	BD Biosciences
CD55	PE	1:400	Biolegend
Podoplanin	Alexa Fluor 488	1:400	Biolegend
CXCL12	APC	5μl	R&D
Live/dead	DAPI/BV510	1:800	Thermo Fisher Scientific
Streptavidin	Brilliant Violet 605	1:200	Biolegend

Supplementary Table 2: Target sequences of CD90, CD55 and CXCL12

Gene symbol	TRC Id	Target sequence
CD90	TRCN0000054891	GTATAGAGACAAGCTGGTCAA
	TRCN0000054892	GCGAATCCCATGAGCTCCAAT
CD55	TRCN0000067573	CCCAACGAAGAAACCAACAAT
	TRCN0000067575	GTCATCCAATAAGAACATCTA
CXCL12	TRCN0000196073	GCCAACGTCAAGCATCTGAAA
	TRCN0000184347	GATCCAAGAGTACCTGGAGAA

Supplementary Table 3: The sequences of qPCR primers

Genes	Forward	Reverse
β-actin	AGGTCATCACTATTGGCAACGA	CCAAGAAGGAAGGCTGGAAAA
Cd55	ACCTCCACTCCCAGGAAAAG	TAGAGGAGACACCGACTAGCC
Cxcl12	TGCATCAGTGACGGTAAACCA	CACAGTTTGGAGTGTTGAGGAT
Cd90	TGCTCTCAGTCTTGCAGGTG	TGGATGGAGTTATCCTTGGTGTT
Il-6	GAGGATACCACTCCCAACAGACC	AAGTGCATCATCGTTGTTCATACA
Il-1β	AAGGGGACATTAGGCAGCAC	ATGAAAGACCTCAGTGCGGG
Sma	GTCCCAGACATCAGGGAGTAA	TCGGATACTTCAGCGTCAGGA
Tgfb1	CAACAATTCCTGGCGTTACC	TGCTGTCACAAGAGCAGTGA
Il-11	GTTTACAGCTCTTGATGTCTC	GAGTCTTTAACAACAGCAGG
Cxcl1	ACTGCACCCAAACCGAAGTC	TGGGGACACCTTTTAGCATCTT
Cxcl5	CCGCTGGCATTTCTGTTGCTGT	CAGGGATCACCTCCAAATTAGCG
Ccl2	GGTCCCTGTCATGCTTCTGG	GAGTAGCAGCAGGTGAGTGG
Ptp4a2	AGCCCCTGTGGAGATCTCTT	AGCATCACAAACTCGAACCA

Supplementary Table 4: Antibodies for flow cytometry analysis

Antibodies	Fluorchrome	Dilution	Manufacturer
CD8	Alexa Fluor 700	1:100	Thermo Fisher Scientific
CD62L	BV421	1:100	Biolegend
CD4	Per-Cy5	1:100	BD Biosciences
Live/dead	Zombie Aqua	1:800	Thermo Fisher Scientific

Supplementary Table 5: Numbers of stromal cells in GSE114374 and GSE134809

Numbers of stromal cells in GSE114374

	CXCL12 positive	CXCL12 negative	Total	p value
**Control**	2382 (0,73)	902 (0,27)	3284 (1)	< 0,0001
**UC**	1387 (0,49)	1422 (0,51)	2809 (1)	

	CD55 positive	CD55 negative	Total	p value
**Control**	829 (0,25)	2455 (0,75)	3284 (1)	< 0,0001
**UC**	1103 (0,39)	1706 (0,61)	2809 (1)	

	CD90 positive	CD90 negative	Total	p value
**Control**	1820 (0,55)	1464 (0,45)	3284 (1)	< 0,0001
**UC**	1875 (0,64)	934 (0,36)	2809 (1)	

	PDPN positive	PDPN negative	Total	p value
**Control**	873 (0,27)	2411 (0,73)	3284 (1)	< 0,0001
**UC**	2214 (0,79)	595 (0,21)	2809 (1)	

	CD73 positive	CD73 negative	Total	p value
**Control**	68 (0,02)	3216 (0,98)	3284 (1)	ns
**UC**	51 (0,02)	2758 (0,98)	2809 (1)	

Numbers of stromal cells in GSE134809

	CXCL12 positive	CXCL12 negative	Total	p value
**CD non-inflamed**	531 (0,18)	2359 (0,72)	2890 (1)	0.0035
**CD inflamed**	661 (0,21)	2425 (0,69)	3086 (1)	

	CD55 positive	CD55 negative	Total	p value
**CD non-inflamed**	668 (0,23)	2222 (0,77)	2890 (1)	< 0,0001
**CD inflamed**	899 (0,29)	2187 (0,71)	3086 (1)	

	CD90 positive	CD90 negative	Total	p value
**CD non-inflamed**	164 (0,06)	2726 (0,94)	2890 (1)	< 0,0001
**CD inflamed**	371 (0,12)	2715 (0,88)	3086 (1)	

	PDPN positive	PDPN negative	Total	p value
**CD non-inflamed**	95 (0,03)	2795 (0,97)	2890 (1)	< 0,0001
**CD inflamed**	352 (0,11)	2734 (0,89)	3086 (1)	

	CD73 positive	CD73 negative	Total	p value
**CD non-inflamed**	18 (0,01)	2872 (0,99)	2890 (1)	0.0002
**CD inflamed**	52 (0,02)	3034 (0,98)	3086 (1)	
